# Hypoxia-Challenged sEVs-Engineered Nanofiber Scaffolds Accelerate Diabetic Wound Healing via Reversing Cellular Dysfunction of Skin Repair Cells

**DOI:** 10.34133/research.1248

**Published:** 2026-05-07

**Authors:** Kailu Guo, Junfeng Gong, Weicheng Zhong, Yiqing Zhang, Yangmengyuan Xu, Yaying Hao, Zhan Xu, Liqian Ma, Junli Chen, Yejiao Shi, Xi Liu, Xiaobing Fu, Cuiping Zhang

**Affiliations:** ^1^ College of Graduate, Tianjin Medical University, Tianjin, China.; ^2^ Medical Innovation Research Department, PLA General Hospital, Beijing, China.; ^3^ Chinese PLA Medical School, Beijing, China.; ^4^ PLA Key Laboratory of Tissue Repair and Regenerative Medicine, Beijing, China.; ^5^ Institute of Translational Medicine, Shanghai University, Shanghai 200444, China.

## Abstract

The persistent hyperglycemic microenvironment in diabetic wounds causes dysfunction of repair cells, resulting in impaired angiogenesis and disorganized extracellular matrix (ECM) deposition. Small extracellular vesicles (sEVs) derived from hypoxia-challenged chorionic plate mesenchymal stem cells (CP-MSC-sEVs^Hypo^) have been explored as novel acellular therapeutics. However, the effects of oxygen tension during culturing parent cells on the pro-regenerative efficacy and mechanisms of CP-MSC-sEVs have not been systematically investigated. In addition, their rapid clearance and limited interaction with target cells at wound sites constrained therapeutic efficacy. In this study, oxygen tension was first optimized systematically during the precondition of CP-MSCs to improve the pro-regenerative properties of the derived sEVs^Hypo^. Then, sEVs^Hypo^-engineered nanofiber scaffolds were fabricated to achieve the sustained release of sEVs^Hypo^ at wound sites by polydopamine (PDA)-mediated interfacial adhesion. In vitro experiments revealed that sEVs obtained under 5% oxygen tension (sEVs^Hypo-5%^) exhibited the most pronounced angiogenic and collagen-regenerative performance. Furthermore, small RNA sequencing and bioinformatic analyses revealed that miR-21-5p was the most abundant miRNA in sEVs^Hypo-5%^, and functional rescue assays validated that miR-21-5p was the key mediator of endothelial activation and ECM remodeling. To improve the bioavailability of sEVs^Hypo-5%^, PDA was leveraged to immobilize sEVs^Hypo-5%^ onto a biocompatible, ECM-mimicking poly(ε-caprolactone) nanofiber scaffold to achieve high loading efficacy and sustained release. In a full-thickness diabetic wound model, the sEVs^Hypo-5%^-loaded nanoscaffold accelerated wound closure and achieved superior pro-healing effects on D14 via enhancing neovascularization and ECM deposition, as confirmed by histological and immunofluorescence analyses. Collectively, this sEVs^Hypo-5%^-loaded nanoscaffold achieved efficient immobilization and sustained delivery of sEVs^Hypo-5%^ to promote angiogenesis and matrix repair by endogenously delivering miR-21-5p at diabetic wound sites, with strong potential for clinical translation.

## Introduction

Diabetic wounds have been recognized as one of the typical complications of diabetes, increasing the heavy physical and economic burden on patients. Generally, persistent hyperglycemia and chronic inflammation lead to the cellular dysfunction of skin repair cells, impairing revascularization and extracellular matrix (ECM) deposition [1]. For example, endothelial cells (ECs) and fibroblasts (FBs) are 2 key effector cells in skin repair cells by orchestrating angiogenesis to restore tissue perfusion and governing the deposition, contraction, and remodeling of ECM, respectively [2]. However, the persistent hyperglycemia environment severely compromises ECs’ function, leading to their mitochondrial dysfunction, impaired nitric oxide signaling, and suppressed angiogenic potential [3,4]. Concurrently, FBs undergo oxidative stress-induced senescence and phenotypic transition, exhibiting aberrant collagen deposition and diminished migratory and ECM-remodeling capacity [5]. This dual dysfunction of ECs and FBs, compounded by disrupted crosstalk with immune cells such as macrophages, perpetuates a pathological cycle of “hypoperfusion–hypoxia–chronic inflammation–ECM dysregulation”, thereby locking diabetic wounds in a nonhealing state [6]. Therefore, restoring cellular functions of skin repair cells is expected to promote diabetic wound healing.

Recently, mesenchymal stem cell-derived small extracellular vesicles (MSC-sEVs) have emerged as a promising cell-free alternative owing to their low immunogenicity, low tumorigenicity, and high biosafety, compared with their counterparts. They mediate intercellular communication and modulate recipient-cell transcriptional programs and signaling pathways by shuttling bioactive proteins, lipids, and nucleic acids between cells. Among MSC sources, perinatal MSCs, particularly chorionic plate-derived mesenchymal stem cells (CP-MSCs), exhibit superior immunomodulatory and pro-angiogenic capabilities compared with adult MSCs [7]. Their ethically accessible source, abundance, and batch-to-batch stability make them highly suitable for scalable production and clinical translation of sEVs [8]. Recent advances in MSC immortalization and high-yield vesicle production pipelines further highlight their potential as a robust and renewable source for sEVs [9]. The application of CP-MSC-sEVs in diabetic wound therapy has been preliminarily explored. Functionally, CP-MSC-sEVs have been reported to markedly promote the proliferation, migration, and tube-formation behaviors of ECs and FBs, thereby improving wound-closure rates in diabetic wound models [10]. Consistent with our previous studies, CP-MSC-sEVs markedly accelerated wound closure in diabetic models [11]. Based on these advantages, CP-MSCs were selected as the ideal source of sEVs in this study to maximize therapeutic efficacy for promoting angiogenesis and matrix repair.

Regrettably, the therapeutic efficacy of native sEVs remains limited. Recently, various engineered strategies have been developed to improve the therapeutic or targeting efficacy of sEVs [12]. Among these approaches, modulating the culture conditions of their parental cells is considered as a safe, mild, and efficient strategy, as they avoid genetic manipulation using lentiviral or adenoviral systems. Moreover, they can preserve the membrane’s structural integrity and high bioactivity of sEVs compared with post-isolation modification methods. Hypoxic preconditioning has emerged as a powerful strategy to enhance the pro-healing capacity of sEVs [13]. Compared with sEVs secreted under normoxic conditions, hypoxia-engineered sEVs consistently exhibit superior angiogenic activity and more robust tissue repair efficacy [14]. For example, 2% to 8% O_2_-preconditioned sEVs promote EC proliferation, migration, and tube formation by stabilizing hypoxia-inducible factor-1α (HIF-1α) and activating downstream pro-regenerative signaling networks, including vascular endothelial growth factor (VEGF) and stromal cell-derived factor-1 [15,16]. Furthermore, hypoxia preconditioning orchestrates a profound remodeling of the vesicular cargo, enriching sEVs with pro-angiogenic microRNAs (miRNAs) such as miR-126 and miR-210-5p, and modulating protein content to activate pathways like phosphatidylinositol 3-kinase/protein kinase B/mammalian target of rapamycin (PI3K/AKT/mTOR), thereby amplifying their regenerative efficacy [17,18]. However, the optimal oxygen tension during CP-MSC culturing to maximize the therapeutic efficacy of CP-MSC-derived sEVs has not yet been systematically investigated.

Additionally, in practical applications, free sEVs are rapidly cleared from systemic circulation by the reticuloendothelial system, leading to inadequate retention at wound sites and a short therapeutic duration. To address this limitation, a variety of biomaterial-based scaffolds and delivery systems have been developed to achieve sustained or controlled release of sEVs, including lyophilized porous scaffolds, GelMA-based hydrogels, microneedle patches, and stimuli-responsive hydrogel systems [19,20]. Electrospun nanofiber scaffolds, in particular, have emerged as a versatile delivery platform, providing structural protection, sustained release, and localized enrichment of bioactive sEVs [21]. Their excellent biocompatibility, mechanical stability, and ECM-mimic properties make them a preferred candidate for wound healing and tissue engineering [22]. Furthermore, the incorporation of functional coatings markedly enhanced the immobilization and delivery efficiency of bioactive cargoes, thereby improving their biofunctionality and therapeutic efficacy [23,24].

**Fig. 1.**
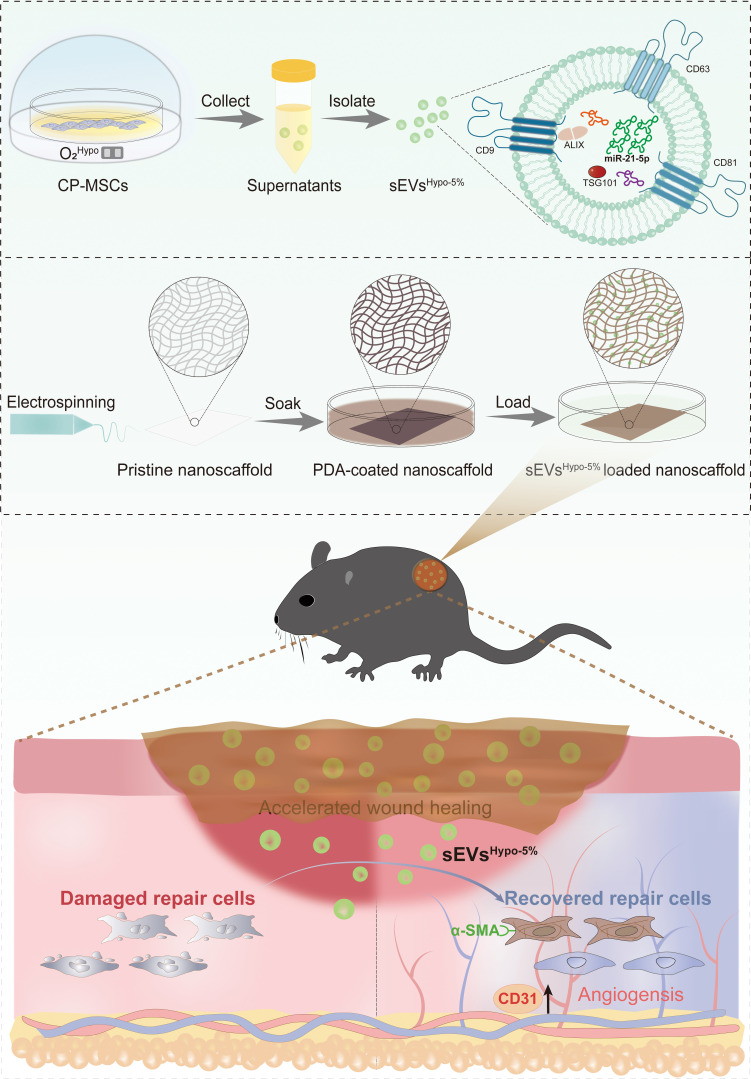
Schematic illustration of the preparation of hypoxia-challenged small extracellular vesicles (sEVs)-engineered nanofiber scaffolds (sEVs^Hypo-5%^-loaded nanoscaffold) and their mechanisms in promoting diabetic wound healing.

Herein, we first systematically investigated the effect of oxygen tension on modulating the pro-regenerative properties of CP-MSC-derived sEVs in high-glucose human umbilical vein endothelial cells (HG-HUVECs) and high-glucose human dermal fibroblasts (HG-HDFs). Then, small RNA sequencing, target prediction, and pathway enrichment analyses were employed to explore the pro-regenerative mechanisms of sEVs^Hypo-5%^. Next, functional rescue assays were performed to validate the critical roles of the identified key miRNA in mediating the pro-angiogenic and pro-collagen regenerative effects of sEVs^Hypo-5%^ on these skin repair cells. To further improve the bioavailability of sEVs^Hypo-5%^, the ECM-mimicking poly(ε-caprolactone) (PCL) nanofibers were fabricated and followed by the polydopamine (PDA) coating to achieve its high loading efficacy and sustained release. Finally, the full-thickness diabetic wound model was established to evaluate the pro-angiogenic effects and collagen deposition-promoting capacity of sEVs^Hypo-5%^-loaded nanoscaffold in vivo. The overall design of the present study is illustrated in Fig. 1.

## Results

### Isolation and characterization of sEVs^Hypo^ and sEVs^Nor^

To identify CP-MSCs used in this study, standard characterization assays were performed prior to hypoxic preconditioning. First, the morphology of CP-MSCs was typically spindle-shaped (Fig. S1A). Second, flow cytometry analysis was employed to examine the expression of typical MSC surface markers. Highly expressed CD90 and CD44 were observed on the surface of CP-MSCs, with low expression of the hematopoietic marker CD45 (Fig. S1B). Thirdly, standard trilineage differentiation assays displayed that CP-MSCs successfully differentiated into adipocytes, osteoblasts, and chondrocytes, further indicating their stemness (Fig. S1C to E).

Next, we explored the effects of 4 commonly used oxygen tensions including 21% O₂ (O₂^Nor^), 5% O₂ (O₂^Hypo-5%^), 3% O₂ (O₂^Hypo-3%^), and 1% O₂ (O₂^Hypo-1%^) on the cell viability of CP-MSCs because the reported oxygen tensions for hypoxic preconditioning of MSCs often varied across studies [25]. As shown in Fig. S2, CP-MSCs maintained their typical spindle-shaped morphology with well-spread cytoplasm, and no apparent cell death or detachment under O₂^Nor^, O₂^Hypo-5%^, and O₂^Hypo-3%^ conditions. In contrast, O₂^Hypo-1%^ induced obvious morphological abnormalities and cell death of CP-MSCs. 5-ethynyl-2ʹ-deoxyuridine (EdU) assays further revealed that CP-MSCs cultured under O₂^Hypo-5%^ exhibited the most proliferative activities, whereas the proliferation behavior was obviously suppressed under O₂^Hypo-1%^ conditions (Fig. S3). These results indicated that moderate hypoxia could maintain or even slightly enhance CP-MSC proliferation, while severe hypoxia (O₂^Hypo-1%^) significantly impaired their proliferative capacity. Therefore, O₂^Nor^, O₂^Hypo-5%^, and O₂^Hypo-3%^ conditions were selected for subsequent hypoxic challenge.

**Fig. 2.**
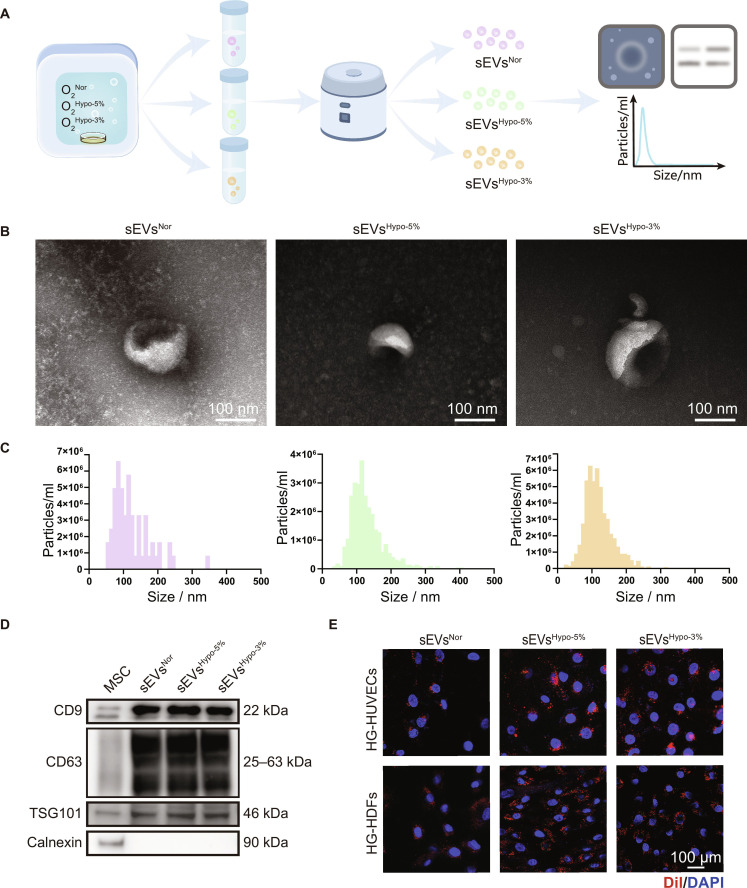
Isolation and characterization of chorionic plate mesenchymal stem cell (CP-MSC)-derived sEVs. (A) Schematic showing the isolation and characterization of sEVs derived from CP-MSCs cultured under O₂^Nor^, O₂^Hypo-5%^, or O₂^Hypo-3%^ conditions. (B) TEM images showing the typical bilayer structure of sEVs^Nor^, sEVs^Hypo-5%^, and sEVs^Hypo-3%^; scale bar = 100 nm. (C) Nanoparticle tracking analysis (NTA) profiles showing the size distribution and concentration of sEVs^Nor^, sEVs^Hypo-5%^, and sEVs^Hypo-3%^ after 400-fold dilution. (D) Western blotting analysis showing the expression of CD9, CD63, TSG101, and Calnexin proteins in these 3 sEVs. (E) Confocal microscopy images showing the internalization of Dil-labeled sEVs by high-glucose human umbilical vein endothelial cells (HG-HUVECs) and high-glucose human dermal fibroblasts (HG-HDFs); scale bar = 100 μm.

After culturing CP-MSCs under O₂^Nor^, O₂^Hypo-5%^, and O₂^Hypo-3%^, sEVs were isolated using a standardized kit and designated as sEVs^Nor^, sEVs^Hypo-5%^, and sEVs^Hypo-3%^ (Fig. 2A). Transmission electron microscopy (TEM) images revealed that they all exhibited the characteristic cup-shaped, bilayer membrane morphology of sEVs (Fig. 2B). Nanoparticle tracking analysis (NTA) results showed that no significant differences were observed among these 3 sEVs regarding the average diameter and particle concentrations (Fig. 2C). Specifically, the average diameters of sEVs^Nor^, sEVs^Hypo-5%^, and sEVs^Hypo-3%^ were 124.5, 130.6, and 120.1 nm, respectively. The particle concentrations of sEVs^Nor^, sEVs^Hypo-5%^, and sEVs^Hypo-3%^ were 9.9 × 10^9^, 1.1 × 10^10^, and 2.0 × 10^10^ particles/ml, respectively. Meanwhile, canonical sEV markers including CD9, CD63, and TSG101 were present in these 3 sEVs, whereas Calnexin was absent, indicating the purity of the isolated sEVs (Fig. 2D). Then, we incubated Dil-labeled sEVs with HG-HUVECs and HG-HDFs for 6 h to observe their internalization in target cells. As shown in Fig. 2E, the red fluorescence of sEVs was distributed around the nuclei of HG-HUVECs and HG-HDFs, indicating their successful cellular internalization. Collectively, these results demonstrated the successful isolation of these 3 sEVs and they could be readily internalized by HG-HUVECs and HG-HDFs.

### Hypoxia-challenged sEVs reverse cellular dysfunction in HG-HUVECs

During the healing process, the formation of granulation tissue generally indicates the transition from the inflammatory phase to the proliferative phase, which primarily involves ECs and FBs. However, under a hyperglycemic microenvironment, excessive ROS production accompanied by advanced glycation end products–receptor for advanced glycation end products-mediated amplification of inflammatory signaling impairs the proliferative and migratory capacities of ECs and FBs. Accordingly, in the following sections, we systematically investigated the effects of these 3 sEVs on restoring the cellular function in HG-HUVECs and HG-HDFs.

**Fig. 3.**
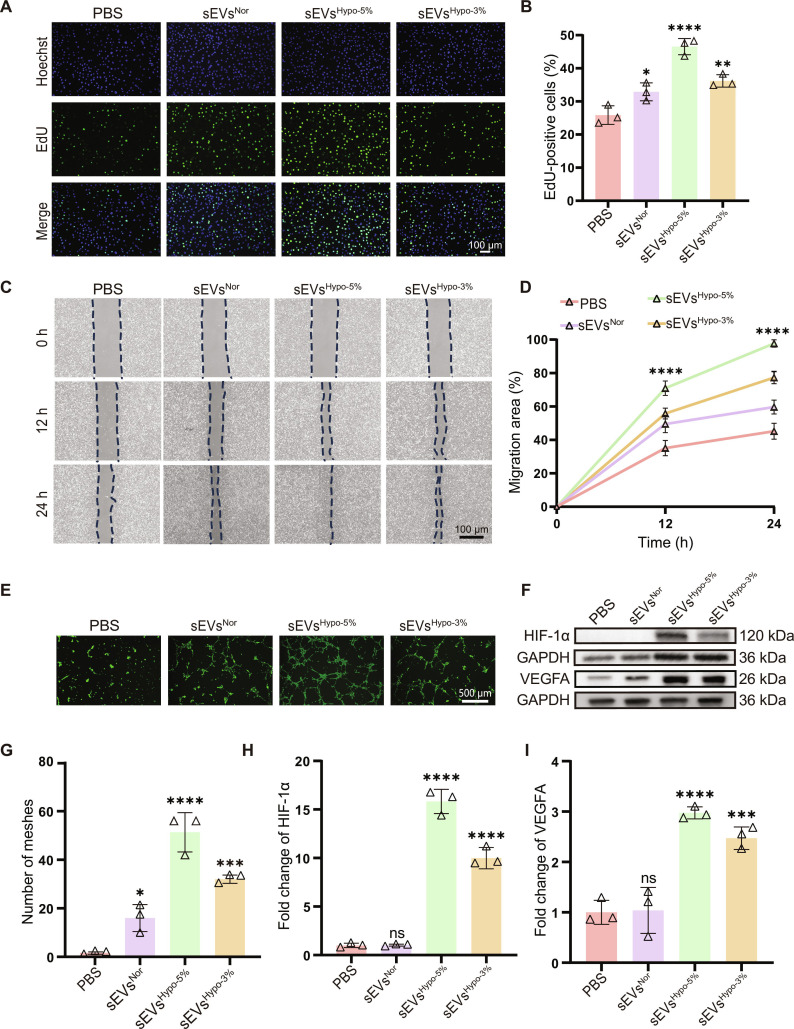
Hypoxia-challenged sEVs reverse cellular dysfunction in HG-HUVECs. (A) Representative EdU images showing the proliferation behaviors of HG-HUVECs treated with PBS, sEVs^Nor^, sEVs^Hypo-5%^, or sEVs^Hypo-3%^; scale bar = 100 μm. (B) Quantification analysis of EdU-positive cells in (A). (C) Scratch-wound healing assay evaluating the migration behaviors of HG-HUVECs under different treatments; scale bar = 100 μm. (D) Quantification of wound closure area in (C). (E) Matrigel tube-formation assay assessing the angiogenic potential of HG-HUVECs in response to sEVs treatments; scale bar = 500 μm. (F) Western blot analysis of hypoxia-inducible factor-1α (HIF-1α) and vascular endothelial growth factor A (VEGFA) expression in HG-HUVECs after incubation with different sEVs. (G) Quantification of the number of meshes in (E). (H and I) Quantitative analysis of HIF-1α and VEGFA protein expression in (F). ns, not significant; *P < 0.05, **P < 0.01, ***P < 0.001, ****P < 0.0001. *n* = 3 per group.

First, Cell Counting Kit-8 (CCK-8) assay was employed to evaluate the cytotoxicity of sEVs on HG-HUVECs. As shown in Fig. S4, these sEVs exhibited no obvious cytotoxicity within the concentration range of 1 × 10^8^ to 1 × 10^10^ particles/ml. Interestingly, the viability of HG-HUVECs increased in a dose-dependent manner. Therefore, 1 × 10^10^ particles/ml was selected as the treatment concentration for subsequent experiments. Then, we examined the effects of these 3 sEVs on restoring HG-HUVECs functions, including proliferation, migration, tube-formation behaviors, and the expression levels of intracellular HIF-1α and VEGFA proteins. Compared with phosphate-buffered saline (PBS), all sEVs obviously increased the proportion of EdU-positive cells in HG-HUVECs, and the most pronounced effect was observed in the sEVs^Hypo-5%^ group, followed by sEVs^Hypo-3%^ and sEVs^Nor^ (Fig. 3A and B). Consistent with the EdU assay, scratch assay revealed that the migration area was the largest in sEVs^Hypo-5%^-treated HG-HUVECs, achieving nearly complete wound closure within 24 h. In contrast, HG-HUVECs treated with sEVs^Nor^ or sEVs^Hypo-3%^ displayed slower migration rates, with the PBS group showing notably weaker migration, further underscoring the enhanced migratory potential of sEVs^Hypo-5%^-treated HG-HUVECs (Fig. 3C and D). Next, Matrigel tube-formation assay was performed to evaluate the angiogenic potential of sEVs-treated HG-HUVECs. As shown in Fig. 3E and G, the markedly increased number of interconnected mesh-like structure was observed in sEVs^Hypo-5%^-treated HG-HUVECs, compared with sEVs^Hypo-3%^ or sEVs^Nor^-treated ones, verifying the superior pro-angiogenic capacity of sEVs^Hypo-5%^. In addition, we also tested the expression of angiogenesis-related proteins including HIF-1α and VEGFA in HG-HUVECs. Western blot analysis showed that the expression levels of HIF-1α and VEGFA proteins were the most up-regulated in HG-HUVECs treated with sEVs^Hypo-5%^, with approximately 15-fold and 3-fold increases, respectively, compared with the PBS control (Fig. 3F, H, and I), indicating that sEVs^Hypo-5%^ activated key angiogenesis-related signaling pathways to promote EC function. Collectively, these results revealed that sEVs^Hypo-5%^ showed the most pronounced effects to restore cellular function in HG-HUVECs.

### Hypoxia-challenged sEVs reverse cellular dysfunction in HG-HDFs

**Fig. 4.**
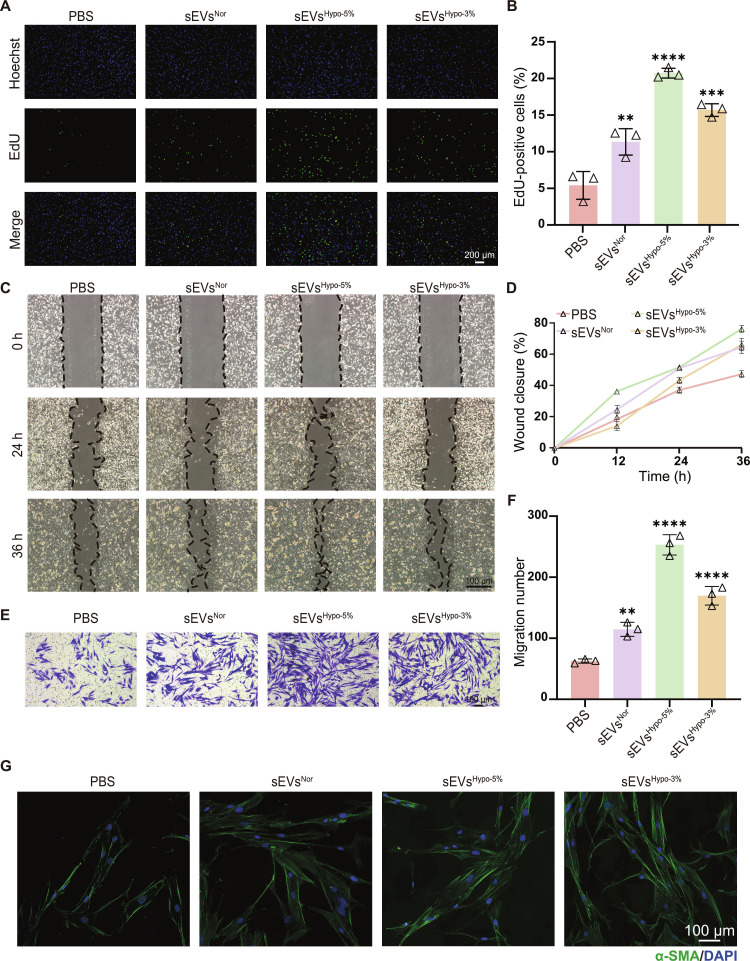
Hypoxia-challenged sEVs reverse cellular dysfunction in HG-HDFs. (A) Representative EdU images showing the proliferation behaviors of HG-HDFs treated with PBS, sEVs^Nor^, sEVs^Hypo-5%^, or sEVs^Hypo-3%^; scale bar = 200 μm. (B) Quantification analysis of EdU-positive cells in (A). (C) Scratch-wound healing assay evaluating the migration behaviors of HG-HDFs under different treatments; scale bar = 100 μm. (D) Quantification of wound closure area in (C). (E) Transwell migration assays for HG-HDFs treated with PBS, sEVs^Nor^, sEVs^Hypo-5%^, or sEVs^Hypo-3%^; scale bar = 100 μm. (F) The number of migration cells in (E). (G) Confocal images of α-smooth muscle actin (α-SMA) immunofluorescence staining in HG-HDFs; scale bar = 100 μm. ns, not significant; *P < 0.05, **P < 0.01, ***P < 0.001, ****P < 0.0001. *n* = 3 per group.

In this section, CCK-8 assays were first employed to evaluate the cytotoxicity of sEVs on HG-HDFs. As shown in Fig. S5, these sEVs exhibited minimal cytotoxicity within the concentration range of 1 × 10^8^ to 1 × 10^10^ particles/ml. Then, the effects of sEVs on reversing HG-HDF dysfunction were investigated. The pro-proliferative function of these 3 sEVs on HG-HDFs was assessed using EdU assays (Fig. 4A and B). Similar to the results in HG-HUVECs, sEVs^Hypo-5%^ also significantly enhanced HG-HDFs proliferation, demonstrating its strong regenerative effect. In scratch assays, HG-HDFs treated with sEVs^Hypo-5%^ exhibited the highest migration rate with the scratched area nearly closed at 36 h. The migration rate of sEVs^Hypo-3%^-treated HG-HDFs and sEVs^Nor^-treated ones was approximately the same (Fig. 4C and D). Transwell assays further confirmed that sEVs^Hypo-5%^ significantly promoted the migration of HG-HDFs, with a markedly higher number of crystal violet-positive ones migrating through the membrane compared with other groups (Fig. 4E and F). Additionally, α-smooth muscle actin (α-SMA; the typical marker of myofibroblasts) immunofluorescence staining indicated that sEVs^Hypo-5%^ promoted the trans-differentiation from FB to myofibroblast, which was the key step in granulation tissue formation and wound contraction (Fig. 4G). Therefore, these findings collectively highlighted that the sEVs^Hypo-5%^ showed the most pronounced effects to restore cellular function in HG-HDFs and sEVs^Hypo-5%^ was selected in succeeding experiments.

### MiR-21-5p is identified as the key mediator of sEVs^Hypo-5%^

**Fig. 5.**
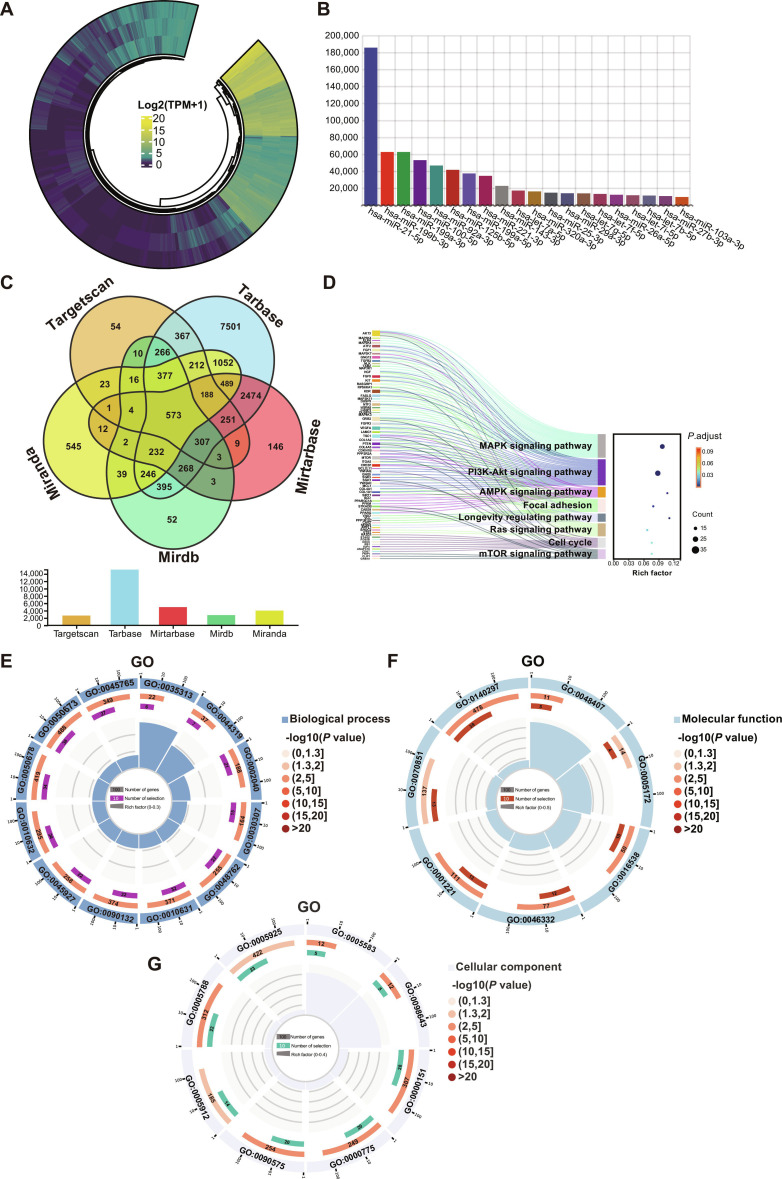
MiRNA sequencing and target gene cluster analysis of sEVs^Hypo-5%^. (A) Circular heatmap showing the overall miRNA expression profiles of sEVs^Hypo-5%^. (B) Bar chart displaying the top 20 most abundant miRNAs identified in sEVs^Hypo-5%^. (C) Venn diagram representing the overlapping target genes of the highly expressed miRNAs predicted or validated across 5 databases (TargetScan, TarBase, miRDB, miRTarBase, and miRanda). (D) Sankey-bubble diagram illustrating the association between target genes and representative signaling pathways, including mitogen-activated protein kinase (MAPK), phosphatidylinositol 3-kinase–protein kinase B (PI3K-AKT), AMP-activated protein kinase (AMPK), Focal adhesion, Longevity regulating, Ras, Cell cycle, and mammalian target of rapamycin (mTOR) pathways. (E to G) Gene Ontology (GO) enrichment analysis of the target genes, categorized into biological process (BP), molecular function (MF), and cellular component (CC) terms.

miRNAs are critically active components within sEVs and play significant roles in regulating cellular functions of skin repair cells, thereby accelerating wound healing [26]. Herein, miRNA sequencing analysis with high reproducibility across replicates was conducted to reveal pronounced pro-healing mechanisms of sEVs^Hypo-5%^ (Fig. 5A). Next, the top 20 enriched miRNAs ranked by logFC value were identified in sEVs^Hypo-5%^ in which miR-21-5p was the most abundant (Fig. 5B). Further, miR-21-5p levels in sEVs^Nor^, sEVs^Hypo-5%^, and sEVs^Hypo-3%^ were measured by quantitative reverse transcription polymerase chain reaction (qRT-PCR). As shown in Fig. S6, miR-21-5p expression was significantly higher in sEVs^Hypo-5%^ than that in sEVs^Nor^ and sEVs^Hypo-3%^. The level of miR-21-5p in sEVs^Hypo-5%^ and sEVs^Hypo-3%^ was 2.43- and 1.87-fold compared to that in sEVs^Nor^.

Then, miRNAs were subjected to Kyoto Encyclopedia of Genes and Genomes (KEGG) signaling pathway enrichment analysis and Gene Ontology (GO) functional clustering analysis. Target genes predicted from 5 databases (TargetScan, TarBase, miRTarBase, miRanda, and miRDB) yielded 573 overlapping genes (Fig. 5C). Furthermore, KEGG pathway analysis revealed that the predicted target genes were significantly enriched in pathways associated with cell proliferation and angiogenesis, including mitogen-activated protein kinase (MAPK) signaling, PI3K–AKT signaling, AMP-activated protein kinase (AMPK) signaling, focal adhesion, Ras signaling, and mTOR signaling pathways. These pathways governed endothelial activation, cytoskeletal remodeling, metabolic regulation, and ECM dynamics during the wound-healing process (Fig. 5D). The GO annotation categorized these genes into biological process (BP), molecular function (MF), and cellular component (CC) domains. The most enriched BP terms were “wound healing, spreading of epidermal cells”, “wound healing, spreading of cells”, and “sprouting angiogenesis”, all of which are essential for endothelial migration, granulation-tissue formation, and vascular sprouting during tissue repair. In the MF category, the top enriched terms included “platelet-derived growth factor binding”, “vascular endothelial growth factor receptor binding”, and “cyclin-dependent protein kinase regulator activity”, indicating regulatory involvement in platelet-derived growth factor- and VEGF-related signaling as well as cell cycle progression. For the CC domain, “fibrillar collagen trimer”, “band 3 complex”, and “chromosome, centromeric region” were the most enriched, reflecting ECM organization and structural components related to active cell proliferation (Fig. 5E to G). Collectively, these results demonstrated that sEVs^Hypo-5%^ harbored a distinct pro-angiogenic and pro-regenerative miRNA signature whose downstream gene networks converged on key signaling pathways regulating neovascularization and matrix remodeling.

To confirm the transfection efficiency, qRT-PCR was performed to measure intracellular miR-21-5p levels in HG-HUVECs and HG-HDFs following mimic transfection. As shown in Fig. S7, miR-21-5p expression was significantly increased in cells treated with sEVs^Hypo-5%^ or miR-21-5p mimic. In contrast, miR-21-5p levels were markedly reduced with the treatment of inhibitor^miR-21-5p^.

**Fig. 6.**
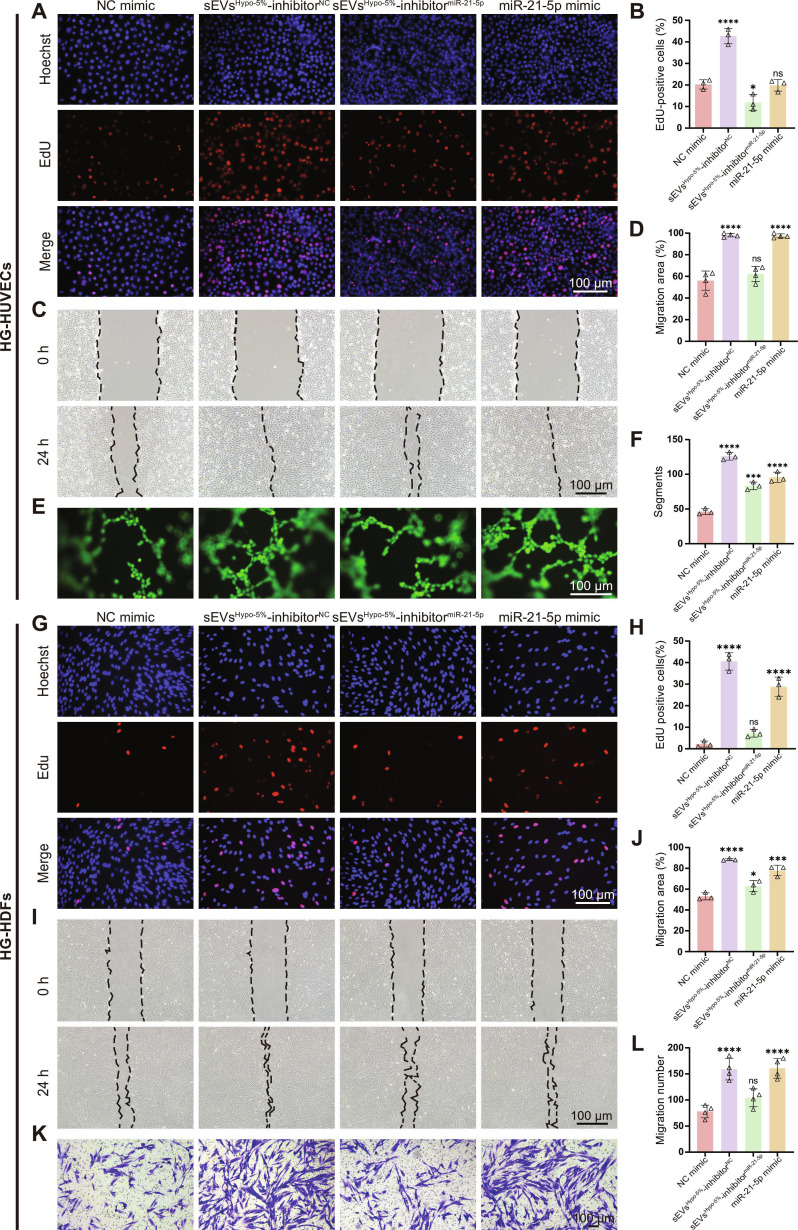
miR-21-5p is the key mediator of sEVs^Hypo-5%^ in recovering cellular function of HG-HUVECs and HG-HDFs. (A) Representative EdU images showing the proliferation of HG-HUVECs treated with NC mimic, sEVs^Hypo-5%^ + inhibitor^NC^, sEVs^Hypo-5%^ + inhibitor^miR-21-5p^, or miR-21-5p mimic. Scale bar = 100 μm. (B) Quantification of (A) (*n* = 3 per group). (C) Scratch wound-healing assay of HG-HUVECs in different groups. Scale bar = 100 μm. (D) Quantification analysis of (C) (*n* = 4 per group). (E) Matrigel tube-formation assay of HG-HUVECs after different treatments. Scale bar = 100 μm. (F) Quantification analysis of segments in (E) (*n* = 3 per group). (G) Representative EdU images showing the proliferation of HG-HDFs treated with NC mimic, sEVs^Hypo-5%^ + inhibitor^NC^, sEVs^Hypo-5%^ + inhibitor^miR-21-5p^, or miR-21-5p mimic. Scale bar = 100 μm. (H) Quantification analysis of (G) (*n* = 3 per group). (I) Scratch wound-healing assay of HG-HDFs. Scale bar = 100 μm. (J) Quantification analysis of (I) (*n* = 3 per group). (K) Transwell migration assay of HG-HDFs after different treatments. Scale bar = 100 μm. (L) Quantification analysis of (K) (*n* = 4 per group). ns, not significant; *P < 0.05, **P < 0.01, ***P < 0.001, ****P < 0.0001.

To validate whether miR-21-5p mediates the pro-angiogenic and pro-regenerative effects of sEVs^Hypo-5%^, a miR-21-5p rescue experiment was performed in HG-HUVECs and HG-HDFs. Cells were treated with NC mimic, sEVs^Hypo-5%^ + inhibitor^NC^, sEVs^Hypo-5%^ + inhibitor^miR-21-5p^, or miR-21-5p mimic, followed by EdU, scratch assay, Transwell, and Matrigel tube-formation assays. EdU staining showed that sEVs^Hypo-5%^ + inhibitor^NC^ and miR-21-5p mimic increased HG-HUVEC proliferation, whereas inhibition of miR-21-5p largely abolished this effect (Fig. 6A and B). In parallel, scratch assays demonstrated enhanced migration in the sEVs^Hypo-5%^ + inhibitor^NC^ and miR-21-5p mimic groups, which was suppressed when miR-21-5p was inhibited by inhibitor^miR-21-5p^ (Fig. 6C and D). Consistently, sEVs^Hypo-5%^ promoted capillary-like tube formation on Matrigel, while miR-21-5p inhibition reduced tube network formation. Conversely, transfection with miR-21-5p mimic increased vascular network formation (Fig. 6E and F). Collectively, these results indicate that miR-21-5p is a critical mediator of sEVs^Hypo-5%^ in restoring cellular functions of HG-HUVECs.

We next examined the critical roles of miR-21-5p in recovering cellular function of HG-HDFs. EdU staining showed that sEVs^Hypo-5%^ + inhibitor^NC^ and miR-21-5p mimic increased HG-HDF proliferation, whereas the inhibition of miR-21-5p largely abrogated this effect (Fig. 6G and H). In addition, scratch assays demonstrated accelerated wound closure in the sEVs^Hypo-5%^ + inhibitor^NC^ group, which was weakened after miR-21-5p inhibition (Fig. 6I and J). Transwell migration assays further confirmed that sEVs^Hypo-5%^ enhanced HG-HDF migration, and miR-21-5p mimic transfection produced a comparable pro-migratory phenotype (Fig. 6K and L). In addition, the effect of miR-21-5p mimic on the expression of α-SMA in HG-HDFs was further investigated. The results showed that the groups of miR-21-5p mimic and sEVs^Hypo-5%^ + inhibitor^NC^ markedly increased α-SMA expression in HG-HDFs, whereas inhibition of miR-21-5p with the inhibitor^miR-21-5p^ attenuated α-SMA expression (Fig. S8). Collectively, these findings indicate that miR-21-5p contributes to the pro-angiogenic and regenerative effects of sEVs^Hypo-5%^ in HG-HUVECs and HG-HDFs.

### Fabrication and characterization of sEVs^Hypo-5%^-loaded nanoscaffold

**Fig. 7.**
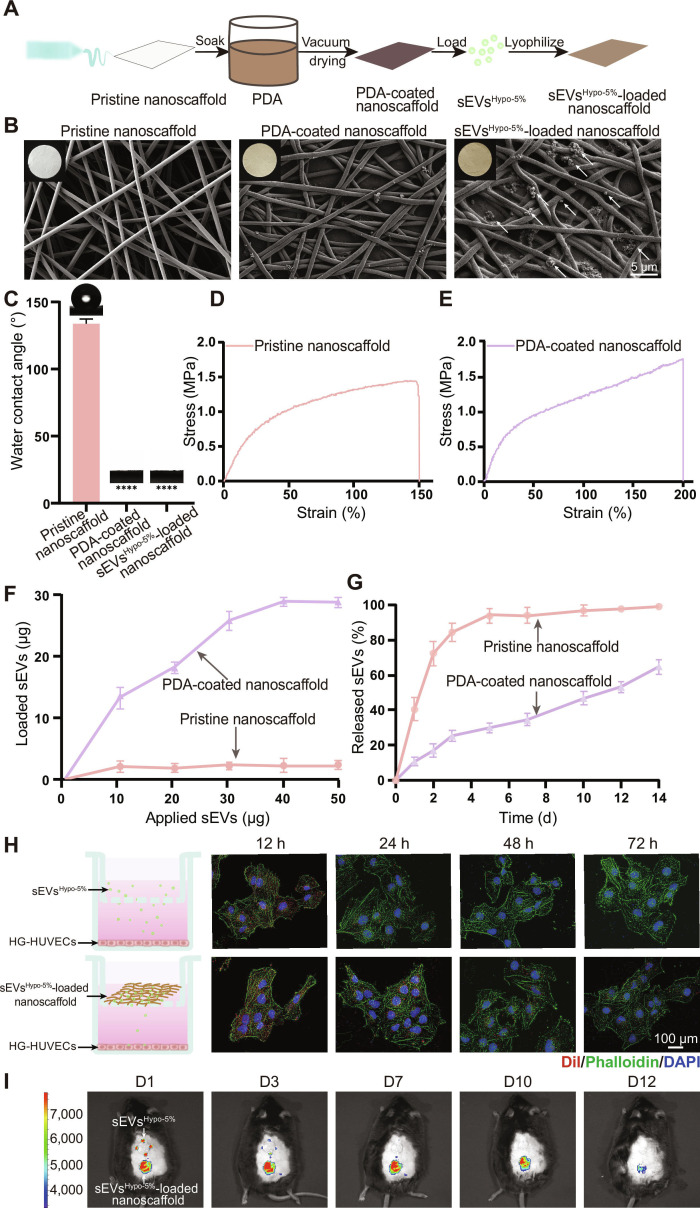
Fabrication and characterization of sEVs^Hypo-5%^-loaded nanoscaffold. (A) Schematics illustrating the preparation process of sEVs^Hypo-5%^-loaded nanoscaffold. (B) SEM images and the optical images of the pristine nanoscaffold, PDA-coated nanoscaffold, and sEVs^Hypo-5%^-loaded nanoscaffold. White arrows indicated the adhesion of sEVs^Hypo-5%^ particles on the surface of PDA-coated nanofibers. Scale bar = 5 μm. (C) Representative images and quantitative analysis of water contact angle of the pristine nanoscaffold, PDA-coated nanoscaffold, and sEVs^Hypo-5%^-loaded nanoscaffold. (D and E) Tensile testing curves evaluating the mechanical strength and flexibility of the pristine nanoscaffold and PDA-coated nanoscaffold. (F) Quantitative BCA assay analysis showing the loading efficiency of sEVs^Hypo-5%^ on the pristine nanoscaffold or PDA-coated nanoscaffold. (G) In vitro release profiles of sEVs from the pristine nanoscaffold and PDA-coated nanoscaffold over 14 d. (H) Illustration and representative fluorescent images reflecting the release profiles of sEVs^Hypo-5%^ in free form or absorbed on the PDA-coated nanoscaffold when incubating with HG-HUVECs; scale bar = 100 μm. (I) In vivo imaging system (IVIS)-based analysis showing the release kinetics of free sEVs^Hypo-5%^ and sEVs^Hypo-5%^ on the PDA-coated nanoscaffold at rodent diabetic wounds. ns, not significant; *P < 0.05, **P < 0.01, ***P < 0.001, ****P < 0.0001.

In practical clinical applications, the bioavailability of free sEVs was relatively low because of their easy inactivation and diffusion around the wounds, reducing their therapeutic efficiency [27]. Therefore, to achieve the sustained release of sEVs^Hypo-5%^ and increase their therapeutic efficacy, dopamine (DA) was self-assembled on ECM-mimicking PCL nanofibers to load sEVs^Hypo-5%^ with high loading efficiency (sEVs^Hypo-5%^-loaded nanoscaffold) by virtue of the strong interfacial adhesion of PDA (Fig. 7A) [28]. As shown in Fig. 7B, the white color of the pristine PCL nanoscaffold was changed to brownish yellow color with the polymerization of PDA, and the loading of sEVs^Hypo-5%^ showed limited effect on the brownish yellow color of the PDA-coated nanoscaffold. Scanning electron microscopy (SEM) images revealed the uniformly distributed fibers within the pristine PCL nanoscaffold with an average diameter of approximately 0.95 μm (Fig. 7B and Fig. S9). The PDA-coated nanoscaffold fibers became slightly bent without obvious diameter change after the self-assembly of PDA and subsequent loading of sEVs^Hypo-5%^ due to light swelling of PDA-coated nanoscaffold fibers during the modifying process. Additionally, a large amount of sEVs^Hypo-5%^ could be observed to adhere on the surface and the network of PDA-coated nanoscaffold fibers, indicating their successful loading. Moreover, PDA coating also changed the wettability of PCL fibers (Fig. 7C). The water contact angle of the pristine PCL nanoscaffold was approximately 129.5° ± 1.7°, reflecting its intrinsic hydrophobicity. However, PDA coating sharply decreased the water contact angle from 129.5° ±1.7° to nearly 0° due to the intrinsic hydrophilicity of PDA. The loading of sEVs^Hypo-5%^ did not alter the wettability of the PDA-coated nanoscaffold with the water contact angle of about 0°. We further tested the effect of PDA coating on the mechanical strength of the pristine PCL nanoscaffold. The results showed that the maximum tensile strength of the pristine PCL nanoscaffold was 1.5 MPa and the maximum strain was 150%, while these values became 1.9 MPa and 200% after PDA modification, respectively, indicating that PDA coating enhanced interfiber adhesion and improved mechanical performance (Fig. 7D and E).

Next, we tested the loading efficacy of sEVs^Hypo-5%^ on the pristine PCL nanoscaffold or the PDA-coated nanoscaffold. Quantitative bicinchoninic acid (BCA) analysis demonstrated that the PDA-coated nanoscaffold exhibited markedly higher loading efficacy and plateaued to reach a saturation level of 29.21 μg, whereas that of the pristine PCL nanoscaffold loaded was only 2.407 μg (Fig. 7F), indicating PDA-mediated high loading efficacy of sEVs^Hypo-5%^ on PCL fibers. Next, release assays were employed to investigate release behaviors of sEVs^Hypo-5%^ from the pristine PCL nanoscaffold or the PDA-coated nanoscaffold. As shown in Fig. 7G, 80% of sEVs^Hypo-5%^ was released from the pristine PCL nanoscaffold within 48 h, exhibiting a pronounced burst-release pattern. In contrast, a sustained-release behavior of sEVs^Hypo-5%^ was observed on the PDA-coated nanoscaffold; that is, 70% of sEVs^Hypo-5%^ was released from the PDA-coated nanoscaffold within 14 d. Therefore, the PDA coating significantly enhanced the loading efficiency of sEVs^Hypo-5%^ and enabled its sustained release at wound sites.

Subsequently, in vitro and in vivo experiments were conducted to observe the sustained release of Dil-labeled sEVs^Hypo-5%^ from the scaffolds. Transwell experiments demonstrated that the red fluorescence of sEVs^Hypo-5%^ could be observed obviously after coincubating with HG-HUVECs for 72 h, whereas the red fluorescence of free sEVs^Hypo-5%^ disappeared after 48 h of coincubation (Fig. 7H). Therefore, the existence of the PDA-coated nanoscaffold prolonged interaction time of sEVs^Hypo-5%^ with skin repair cells. Consistently, in vivo fluorescence imaging revealed that free sEVs^Hypo-5%^ injected around the wound exhibited strong fluorescence accumulation at D1, which markedly declined on D3. In contrast, the fluorescence of sEVs^Hypo-5%^ was obvious on D3 and became faded until D12 (Fig. 7I). The PDA-coated nanoscaffold readily adhered to the fresh wound, and a 0.785-cm^2^ membrane was capable of lifting a 23-g mouse demonstrating the strong wet adhesive property of the scaffold (Fig. S10). Collectively, these results indicated that we fabricated sEVs^Hypo-5%^-loaded nanoscaffold successfully and the PDA-coated nanoscaffold could achieve high loading efficiency and sustained release of sEVs^Hypo-5%^ both in vitro and in vivo.

### sEVs^Hypo-5%^-loaded nanoscaffold accelerates diabetic wound healing

**Fig. 8.**
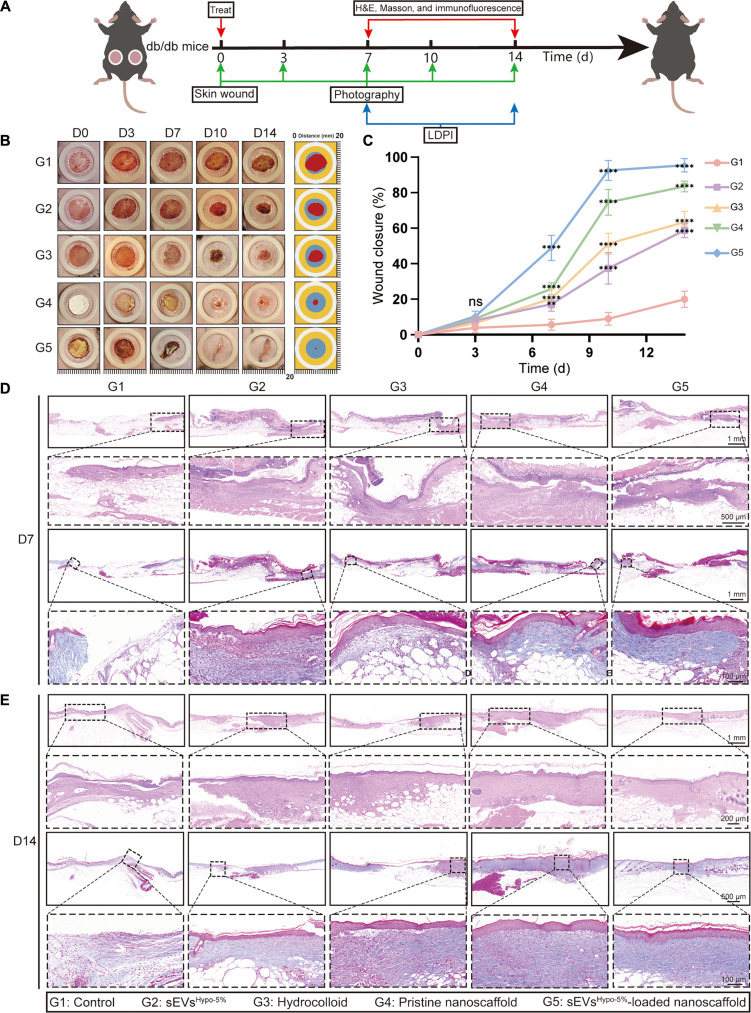
sEVs^Hypo-5%^-loaded nanoscaffold accelerates wound closure and perfusion of diabetic wound. (A) Illustration showing the timeline of animal experiments and the pro-healing effects of sEVs^Hypo-5%^-loaded nanoscaffold on diabetic wounds. (B) Representative wound photographs on D0, 3, 7, 10, and 14. (C) Quantitative wound closure rates over 14 d. (D and E) Hematoxylin and eosin (H&E) and Masson’s trichrome staining of wound tissue sections on D7 and D14. ns, not significant; *P < 0.05, **P < 0.01, ***P < 0.001, ****P < 0.0001. *n* = 4 per group.

Based on the pro-angiogenic and pro-collagen deposition effects of sEVs^Hypo-5%^ in vitro and the successful fabrication of sEVs^Hypo-5%^-loaded nanoscaffold, we next investigated the wound-healing efficacy of sEVs^Hypo-5%^-loaded nanoscaffold in a full-thickness diabetic wound model (Fig. 8A). Mice were randomly assigned to 5 groups and treated with PBS (G1), free sEVs^Hypo-5%^ (G2), commercial hydrocolloid dressing (G3), pristine nanoscaffold (G4), or sEVs^Hypo-5%^-loaded nanoscaffold (G5). Wounds treated with sEVs^Hypo-5%^-loaded nanoscaffold achieved almost complete wound closure on D14, significantly outperforming the other groups (Fig. 8B and C). Wounds treated with free sEVs^Hypo-5%^ injection showed limited healing, with a wound closure rate of 58.8% on D14. The PBS group exhibited the slowest wound closure throughout the observation period. Furthermore, hematoxylin and eosin (H&E) staining revealed extensive re-epithelialization, dense granulation tissue formation, and successful regeneration of skin appendages in wounds treated with sEVs^Hypo-5%^-loaded nanoscaffold. The epithelial thickness was greater than that in the other groups. Additionally, Masson’s trichrome staining showed well-organized collagen deposition and advanced matrix remodeling, further confirming the improved wound quality (Fig. 8D and E). Together, these results demonstrate the potent therapeutic efficacy of sEVs^Hypo-5%^-loaded nanoscaffold in enhancing wound healing.

### sEVs^Hypo-5%^-loaded nanoscaffold enhances neovascularization and ECM deposition in diabetic wounds

**Fig. 9.**
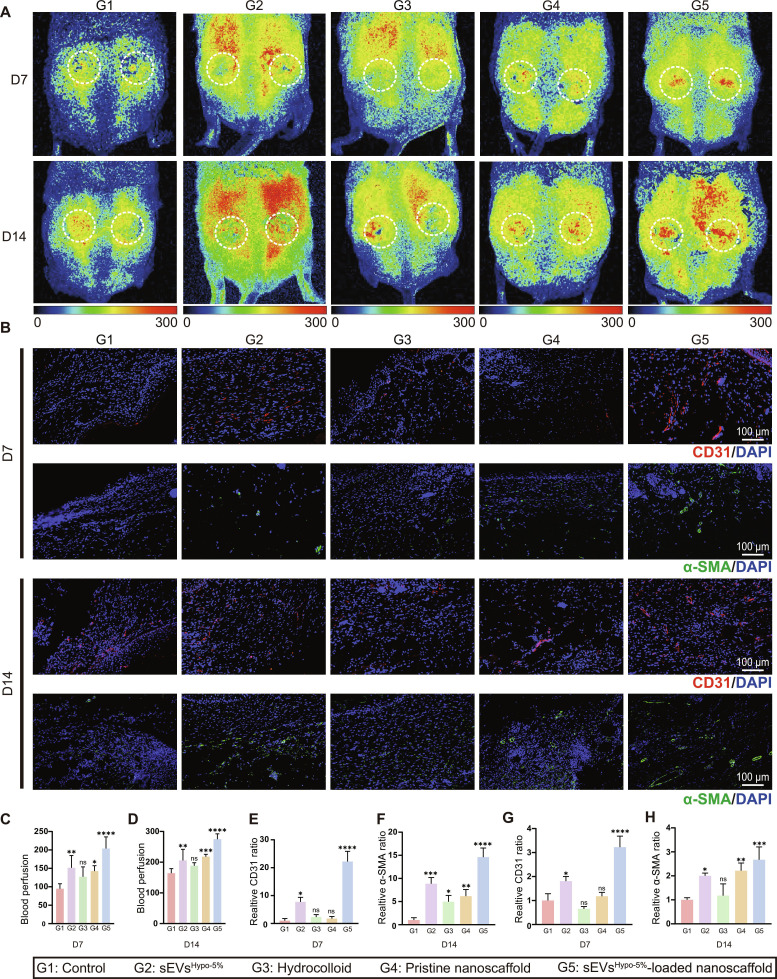
sEVs^Hypo-5%^-loaded nanoscaffold enhances neovascularization and ECM deposition to accelerate diabetic wound healing. (A) Laser Doppler perfusion images showing the blood perfusion of wound beds treated with PBS, free sEVs^Hypo-5%^, hydrocolloid, pristine nanoscaffold, and sEVs^Hypo-5%^-loaded nanoscaffold on D7 and D14. (B) Immunofluorescence staining for CD31 (red) and α-SMA (green) in wound tissue sections at D7 and D14; scale bar = 100 μm. ns, not significant; *P < 0.05, **P < 0.01, ***P < 0.001, ****P < 0.0001. (C and D) Quantitative analysis of relative perfusion intensity in (A). (E to H) Quantitative analysis of (B). ns, not significant; *P < 0.05, **P < 0.01, ***P < 0.001, ****P < 0.0001. *n* = 4 per group.

To further reveal the pro-healing performance of sEVs^Hypo-5%^-loaded nanoscaffold at diabetic wounds, real-time perfusion imaging and immunofluorescence analysis were conducted consecutively. Specifically, the wounds treated with sEVs^Hypo-5%^-loaded nanoscaffold exhibited abundant blood perfusion as early as on D7, whereas those treated with other groups showed obvious blood perfusion until D14, as demonstrated by the real-time perfusion imaging analysis (Fig. 9A, C, and D). Free sEVs^Hypo-5%^ injection resulted in limited perfusion improvement, and the PBS group consistently showed the poorest perfusion. The hydrocolloid dressing and pristine nanoscaffold groups displayed intermediate levels of perfusion recovery. Immunofluorescence staining for proliferating cell nuclear antigen (PCNA) was performed in wound sites with different treatments on D14. The results showed that the sEVs^Hypo-5%^-loaded nanoscaffold-treated wounds exhibited a significantly higher number of PCNA-positive cells, whereas the hydrocolloid and pristine nanoscaffold-treated ones displayed fewer PCNA-positive cells. The control group demonstrated the lowest level of PCNA-positive cells. These results indicated that sEVs^Hypo-5%^-loaded nanoscaffold could facilitate the proliferation of skin repair cells in vivo (Fig. S11).

Then, immunofluorescence analysis was employed to evaluate the expression levels of CD31 (the marker of ECs) and α-SMA (a marker of myofibroblasts) at wound sites treated with the above groups. Consistent with the perfusion imaging results, the expression of CD31 at wounds treated with sEVs^Hypo-5%^-loaded nanoscaffold was the highest at D7 and D14. In particular, CD31 exhibited a tubular distribution pattern as early as on D7 and the obvious tubular distribution pattern on D14, further indicating the occurrence of early neovascularization at sEVs^Hypo-5%^-loaded nanoscaffold-treated wounds (Fig. 9B). Similarly, the expression of α-SMA was also the highest at sEVs^Hypo-5%^-loaded nanoscaffold-treated wounds both on D7 and D14, confirming the activation of FB toward myofibroblasts (Fig. 9E to H). Additionally, the elevated expression of α-SMA also indicated the vascular maturation at sEVs^Hypo-5%^-loaded nanoscaffold-treated wounds, as demonstrated by its tubular distribution pattern. Collectively, sEVs^Hypo-5%^-loaded nanoscaffold accelerated diabetic wound healing by enhancing neovascularization and ECM deposition.

### The biosafety of sEVs^Hypo-5%^-loaded nanoscaffold in vitro and in vivo

High biosafety is a prerequisite for wound treatment and clinical translation of the sEVs^Hypo-5%^-loaded nanoscaffold. At the cellular level, cytocompatibility was assessed by Live/Dead staining in HG-HUVECs and HG-HDFs. No obvious increase in the number of dead cells was observed in any treatment group compared with the control group, indicating the high cytocompatibility of sEVs^Hypo-5%^-loaded nanoscaffold (Fig. S12).

At the animal level, the hemolysis assay showed the minimal hemolytic activity of sEVs^Hypo-5%^-loaded nanoscaffold, indicating its acceptable blood compatibility (Fig. S13). Systemic safety was evaluated on postoperative day 14. Blood samples were collected for serum biochemical analysis. Alanine aminotransferase (ALT) and aspartate aminotransferase (AST) were used to assess liver function, with urea and creatinine (CREA) for renal function, and creatine kinase-MB (CK-MB) for potential cardiac injury. These results indicated that the treatment did not cause detectable hepatic, renal, or cardiac dysfunction within the healing period of wounds (Fig. S14). In addition, major organs were harvested for histological examination, and no obvious inflammatory infiltration or structural alterations were detected compared with the control group (Fig. S15). Collectively, these results demonstrated the high biosafety of sEVs^Hypo-5%^-loaded nanoscaffold.

## Discussion

The cutaneous repair process is a staged yet interwoven program spanning hemostasis, inflammation, proliferation, and remodeling phases [29]. However, the high-glucose microenvironment of diabetic wounds disrupts this orderly process, resulting in delayed wound closure and compromised tissue regeneration [30]. Specifically, the hyperglycemic microenvironment led to the dysfunction of vascular ECs and FBs, hindering the regeneration of granulation tissue, restricting the progression of the proliferative phase in diabetic wounds [31]. Current therapeutic approaches for diabetic wounds mainly include pharmacological interventions, surgical debridement, and certain biomaterial-based dressings [32,33]. Regrettably, owing to the multifactorial pathophysiology underlying impaired wound healing, these approaches often fail to fundamentally correct the functional dysfunction of cutaneous repair cells, thereby limiting durable and comprehensive tissue regeneration. In the present study, we integrate hypoxia-engineered CP-MSC-sEVs with the PDA-coated nanoscaffold, aiming to achieve sustained delivery and enhanced bioactivity of therapeutic vesicles. This bioengineered construct amplifies angiogenic and matrix-regenerative functions while enabling efficient immobilization and sustained release of sEVs, offering a translationally promising solution for diabetic wound management.

Although several studies have reported the pro-regenerative potential of hypoxia-preconditioned sEVs, the optimal oxygen tension required to maximally enhance their regenerative efficacy remains unclear [34]. Therefore, in this study, we first systematically investigated the impact of oxygen tension on the pro-healing effects of CP-MSC-derived sEVs. We investigated the effects of different oxygen concentrations, including normoxia, 5%, 3%, and 1% O₂, on the viability and proliferation of CP-MSCs. The results showed that when the oxygen concentration decreased to 1%, cell viability significantly decreased and proliferation was essentially halted. Existing studies suggest that extreme hypoxia can lead to mitochondrial metabolic imbalance and excessive ROS, endoplasmic reticulum stress/unfolded protein response activation, as well as up-regulation of cell cycle arrest and apoptotic signaling, thereby inhibiting MSCs survival and expansion [35]. Based on the balance between safety and functionality, in subsequent experiments, we selected 3% and 5% O₂ as hypoxic conditions, with normoxia as the control, to systematically evaluate the differences in therapeutic cargo and efficacy of sEVs.

Next, we investigated the effects of sEVs generated under different oxygen conditions on the restoration of functional impairments in 2 key cell types involved in granulation tissue regeneration, namely, ECs and FBs. We found that sEVs^Hypo-5%^ showed the most significant performance in promoting angiogenesis-related functions, being able to markedly enhance the proliferation, migration, and tube-forming ability of vascular ECs, while simultaneously up-regulating key angiogenic proteins such as HIF-1α and VEGFA, thereby effectively restoring the function of ECs impaired by the high-glucose microenvironment in diabetes.

To investigate why sEVs^Hypo-5%^ exhibited superior pro-regenerative activity, we performed transcriptomic analysis to explore the underlying molecular mechanisms. The results showed that in sEVs^Hypo-5%^, the expression levels of multiple angiogenesis-related miRNAs were significantly increased, among which miR-21-5p was highly expressed in the sEVs^Hypo-5%^. These miRNAs were primarily enriched in classic angiogenesis- and repair-related pathways such as the PI3K/AKT signaling pathway, thereby regulating key processes such as cell proliferation, migration, and anti-apoptosis, providing a molecular-level explanation for the superior reparative performance of sEVs^Hypo-5%^. Next, functional rescue assays demonstrated that blocking miR-21-5p significantly weakened the effects of sEVs^Hypo-5%^ on endothelial and FB proliferation and migration, indicating that miR-21-5p is a major functional mediator of sEVs^Hypo-5%^. Consistent with previous reports, miR-21-5p functions as a key regulator of wound repair by engaging pro-angiogenic signaling pathways such as PI3K/AKT, thereby enhancing endothelial and FB proliferation and migration, promoting angiogenesis, and supporting ECM remodeling [36].

However, in practical application of sEVs, their tendency to diffuse easily and their short retention time will affect the therapeutic effect; if frequent injections are required, it will increase patient pain and reduce patient compliance. Electrospun fibers, owing to their similarity to the ECM, simple preparation, and high design flexibility, have achieved widespread application in the field of tissue regeneration [37]. Accumulating evidence suggests that surface modification of electrospun fibers can further enable their use as effective scaffolds for sEV delivery. For instance, phospholipid-grafted electrospun poly(L-lactic acid) fibers have been employed to immobilize and locally deliver MSC-derived sEVs via membrane intercalation, thereby improving vesicle retention and accelerating diabetic wound healing. Inspired by mussel-adhesive chemistry, catechol-containing materials exhibit strong interfacial adhesion and have been widely adopted for surface functionalization. For instance, PDA-modified electrospun PLLA scaffolds have also been used for sEVs immobilization, enabling sustained release and improved therapeutic performance [38]. Consistently, PDA-functionalized electrospun SF/PCL nanofibers have also been reported to stably anchor MSC-sEVs and preserve their bioactivity in regenerative repair models [39]. Meanwhile, PCL, owing to its excellent biocompatibility and favorable mechanical stability, has been widely used in wound repair, nerve regeneration, and other fields.

Therefore, taking advantage of the strong adhesion of catechol and the ECM-like properties of electrospinning, we constructed the PDA-coated nanoscaffold [40]. The results showed that the introduction of PDA enhanced the adhesion between extracellular vesicles and the fibers, increasing the sEVs loading capacity by more than 2-fold. Moreover, long-term sustained release of sEVs^Hypo-5%^ was achieved both in vitro and in vivo. In addition, our material exhibited high biosafety. This favorable safety profile can be largely attributed to the use of a biocompatible PCL substrate and a mild, biointerface-compatible PDA coating. PCL is a well-established biodegradable polymer that has been extensively applied in biomedical and regenerative medicine fields with a documented safety profile [41]. Meanwhile, PDA has been widely employed as a versatile surface modification strategy for biomedical materials, and is generally reported to exhibit good biocompatibility when deposited under appropriate conditions [42]. Excellent biocompatibility is important for clinical translation of sEVs^Hypo-5%^-loaded nanoscaffold. In this study, the materials used in the scaffold, including PCL, PDA, and CP-MSC-derived sEVs, have been widely reported for their good safety. We also evaluated the cytotoxicity, hemocompatibility, and systemic safety of the sEVs^Hypo-5%^-loaded nanoscaffold. The results showed that the scaffold did not induce detectable cytotoxicity in HG-HUVECs or HG-HDFs. Additionally, sEVs^Hypo-5%^-loaded nanoscaffold also showed acceptable blood compatibility and the minimal toxicity of the liver, heart, and kidneys within the healing period.

Furthermore, the sEVs^Hypo-5%^-loaded nanoscaffold we constructed demonstrated the best wound-healing effect in rodent diabetic wound models. H&E and Masson’s trichrome staining revealed more complete re-epithelialization, enhanced granulation tissue formation, and more abundant, well-organized collagen deposition, indicating accelerated ECM reconstruction and wound healing. In line with these histological findings, real-time perfusion imaging showed obvious blood perfusion as early as D7, suggesting early establishment of functional microcirculation. Immunofluorescence analysis further confirmed robust vascular responses, with elevated CD31 and α-SMA expression at D7 and D14, reflecting enhanced angiogenesis, vascular maturation, and collagen deposition. This superior healing performance can be attributed to the coordinated mechanism. The PCL-based pristine nanoscaffold provides an ECM-mimicking architecture and mechanical support that facilitates cell adhesion, migration, and granulation tissue ingrowth. Surface modification with PDA enables efficient immobilization and sustained local release of sEVs while preserving their bioactivity. In parallel, the continuously delivered sEVs^Hypo-5%^, enriched in pro-angiogenic miRNAs such as miR-21-5p, activates angiogenesis-associated signaling pathways (MAPK, PI3K/AKT, and AMPK), thereby promoting timely neovascularization and orderly matrix remodeling in diabetic wounds.

Therefore, the sEVs^Hypo-5%^-loaded nanoscaffold is expected to become a promising wound dressing. Collectively, this hypoxia-challenged sEVs-integrated scaffold provides a robust acellular strategy for chronic diabetic wound management and may inspire next-generation vesicle-based biomaterials for translational wound therapy.

## Conclusion

In conclusion, sEVs^Hypo-5%^ exhibited the most pronounced angiogenic and collagen-regenerative performance, by endogenously delivering miR-21-5p in HG-HUVECs and HG-HDFs. PDA-coated and ECM-mimic nanoscaffold was leveraged to achieve the high loading efficacy and sustained release of sEVs^Hypo-5%^ both in vitro and in vivo. The fabricated sEVs^Hypo-5%^-loaded nanoscaffold with high biosafety accelerated obvious diabetic wound closure via enhancing neovascularization and ECM deposition, establishing their potential as a promising therapeutic approach for chronic wound management.

## Materials and Methods

### Cell culture

CP-MSCs were kindly provided by Professor Hongyu Sun’s group (Western Theater General Hospital, China) and met the International Society for Cellular Therapy (ISCT) criteria for mesenchymal stem cells (2006). To evaluate the effects of oxygen tension on the characteristics of sEVs secreted by CP-MSCs, cells were cultured under normoxia (O₂^Nor^) or hypoxia (O₂^Hypo-5%^, O₂^Hypo-3%^, or O₂^Hypo-1%^) using a tri-gas incubator (Ox-101C, TOW-INT TECH, China) and a commercially available MSC culture medium (YOUCON, NC1013, China). When the oxygen level was reduced to 1%, CP-MSCs displayed marked abnormalities (numerous floating/dead cells) consistent with previous reports [43]. Because stable proliferation could not be maintained at O₂^Hypo-1%^, subsequent experiments were conducted only between O₂^Nor^, O₂^Hypo-5%^, and O₂^Hypo-3%^. Previous studies similarly demonstrated that severe hypoxia, while capable of activating the HIF-1α pathway, often led to metabolic imbalance, increased apoptosis, and reduced sEVs yield and stability. All groups of CP-MSCs were cultured under standard conditions (37 °C, 5% CO₂). HUVECs (CRL-1730, ATCC, USA) were cultured in DMEM containing 10% fetal bovine serum (FBS), 1% PS, and 50 mM glucose. HDFs (ATCC, CRL-4053, USA) were cultured in DMEM, containing 10% FBS, 1% PS, and 50 mM glucose for 60 d.

### Flow cytometry analysis of surface markers of CP-MSCs

CP-MSCs were harvested and washed twice with PBS. Cells were incubated with fluorochrome-conjugated antibodies against CD90, CD44, and CD45 for 30 min at 4 °C in the dark. After washing, cells were resuspended in PBS and analyzed using a flow cytometer (BD FACS Calibur, Becton-Dickinson, USA).

### Trilineage differentiation assays of CP-MSCs

To assess the multipotent differentiation potential, CP-MSCs were induced toward adipogenic, osteogenic, and chondrogenic lineages by adipogenic differentiation medium (XR-hMADM; XRbio, China), osteogenic differentiation medium (XR-hMODM; XRbio, China), and chondrogenic differentiation medium (XR-hMCDM; XRbio, China), respectively. For adipogenic differentiation, cells were cultured in adipogenic induction medium for 14 d. Lipid droplets were visualized by Oil Red O staining. For osteogenic differentiation, cells were cultured in osteogenic induction medium for 21 d. Mineralized nodules were detected by Alizarin Red S staining. For chondrogenic differentiation, cell pellets were cultured in chondrogenic induction medium for 21 d. Cartilage matrix formation was evaluated by Alcian Blue staining. Stained samples were observed under a light microscope, and representative images were recorded.

### Isolation and characterization of sEVs

CP-MSCs were cultured under O₂^Nor^, O₂^Hypo-5%^, and O₂^Hypo-3%^ conditions for 48 h, and the cell supernatants were collected. sEVs^Nor^, sEVs^Hypo-5%^, and sEVs^Hypo-3%^ were isolated using a commercial exosome purification kit (Ome-01E, Omiget, China) following the manufacturer’s instructions. Briefly, the supernatants were sequentially centrifuged at 300 *g* for 10 min to remove floating cells, followed by 3,000 *g* for 20 min to eliminate cellular debris and larger vesicles, and then subjected to kit-based purification.

The morphology of the purified sEVs was observed using TEM (Hitachi, Japan). NTA (Particle Metrix GmbH, Germany) was performed to determine the size distribution and concentration of sEVs. Western blotting was performed to verify sEVs markers. Primary antibodies against CD9 (13174, Cell Signaling Technology, USA), CD63 (PAB48050, Bioswamp, China), and TSG101 (72312, Cell Signaling Technology, USA) were used as positive markers, while Calnexin (ab92573, Abcam, UK) was used as negative marker.

### sEVs labeling and cellular uptake

sEVs were membrane-labeled with the lipophilic fluorescent dye Dil (C1036, Beyotime, China). Briefly, sEVs (100 μl) were incubated with Dil for 40 to 60 min, followed by ultracentrifugation and subsequent washing steps to remove excess dye molecules. HG-HDFs and HG-HUVECs were seeded in 24-well plates and cultured to approximately 60% confluence, and then incubated with Dil-labeled sEVs for 4 h. After incubation, cells were washed with PBS, counterstained with Hoechst 33342 (C1025, Beyotime) for nuclei and Phalloidin-488 (HY-P0028, MCE, USA) for F-actin, and thereafter imaged using a confocal laser scanning microscope (CLSM; Leica, Germany).

### CCK-8 assays

HG-HUVECs and HG-HDFs were seeded in 96-well plates (5 × 10^3^ cells/well) and allowed to adhere overnight (37 °C, 5% CO₂). The following day, cells were treated with a concentration gradient of sEVs^Hypo-5%^ at final concentrations of 0, 1 × 10^8^, 5 × 10^8^, 1 × 10^9^, 5 × 10^9^, and 1 × 10^10^ particles/ml. Cells were incubated under serum-free conditions for 24 to 48 h to minimize serum interference with sEVs uptake and signaling. At the end of treatment, 10 μl of CCK-8 reagent (CA1210, Solarbio) was added to each well and incubated for an additional 2 h at 37 °C. Absorbance was measured at 450 nm using a microplate spectrophotometer (Bio-Rad 680, USA).

### EdU proliferation assay

HG-HDFs and HG-HUVECs were seeded in 24-well plates at approximately 70% to 80% confluence and incubated with EdU (C0071S, Beyotime) working solution (1:1,000) at 37 °C for 2 h. Cells were then fixed with 4% paraformaldehyde for 15 min, and permeabilized with 0.3% Triton X-100 for 10 min. Nuclei were counterstained with Hoechst 33342, and images were acquired using a fluorescence microscope. EdU-positive rates (% EdU^+^/total) were quantified using ImageJ Cell Counter plugin.

### Scratch assay

HG-HUVECs and HG-HDFs were seeded in 6-well plates and cultured to approximately 90% confluence. A uniform linear scratch was created across the cell monolayer using a sterile 1-ml pipette tip. After scratching, nonadherent cells were removed by PBS, and serum-free medium was added to minimize the interference of cell proliferation with migration assessment. Cells were then treated with PBS, sEVs^Nor^, sEVs^Hypo-5%^, or sEVs^Hypo-3%^ and subsequently incubated at 37 °C and 5% CO₂. Wound closure was imaged at 0, 12, and 24 h post-scratch using an inverted microscope. The wound closure area (%) was quantified using ImageJ software.

### Transwell migration

Directional migration of HG-HDFs was assessed using 8-μm-pore Transwell inserts (polycarbonate, Corning, USA). HG-HDFs were pretreated for 24 h with PBS, sEVs^Nor^, sEVs^Hypo-5%^, or sEVs^Hypo-3%^, and subsequently detached and resuspended in serum-free medium. Cells (2 × 10^4^) were seeded in the upper chamber, while the lower chamber contained 500 μl of complete medium supplemented with 10% FBS as the chemoattractant. After 24 h, nonmigrated cells were removed, and the membranes were washed, fixed with 4% paraformaldehyde for 15 min, and stained with crystal violet for 20 min. Images were acquired, and migrated cells were quantified using ImageJ. Migration rates were normalized to the PBS control group.

### Tube formation

Matrigel (50 μl/well, Corning) was dispensed into precooled 96-well plates and allowed to solidify at 37 °C for 30 min. HG-HUVECs pretreated for 24 h with PBS, sEVs^Nor^, sEVs^Hypo-5%^, or sEVs^Hypo-3%^ were seeded at 1 × 10^4^ cells/well onto Matrigel-coated wells and subsequently incubated for 6 to 8 h at 37 °C and 5% CO₂ to form capillary-like networks. Networks were imaged using phase-contrast microscopy and quantified using ImageJ.

### Western blotting analysis

To investigate the regulatory effects of sEVs on angiogenesis-related signaling pathways in HG-HUVECs, the expression levels of VEGFA and HIF-1α were quantified by Western blotting. HG-HUVECs were seeded in 6-well plates and cultured until 40% to 50% confluence before treatment with PBS, sEVs^Nor^, sEVs^Hypo-5%^, or sEVs^Hypo-3%^. Cells were further incubated until reaching approximately 90% confluence. Total protein was extracted using RIPA lysis buffer (R0010, Solarbio) supplemented with protease and phosphatase inhibitors, and quantified via BCA protein assay. Equivalent amounts of protein were separated by SDS-PAGE and electrotransferred onto polyvinylidene difluoride membranes. After blocking for 1 h at room temperature to minimize nonspecific binding, membranes were incubated overnight at 4 °C with primary antibodies: anti-VEGFA (A12303, ABclonal, China) and anti-HIF-1α (A26889, ABclonal). Subsequently, membranes were incubated with horseradish peroxidase-conjugated secondary antibodies (ZB-2301, ZSGB-BIO, China) for 1 h at room temperature. Immunoreactive protein bands were visualized using enhanced chemiluminescence (BL523B, Biosharp, China) and imaged with a chemiluminescence detection system (Tanon, China). Quantitative analysis of band intensities was performed using ImageJ software and protein expression levels normalized for intersample comparison.

### qRT-PCR analysis

Total RNA from sEVs was extracted using an RNA extraction kit (YI SHAN Biotechnology, China) according to the manufacturer’s instructions. Reverse transcription and quantitative PCR were performed using the miDETECT A Track miRNA qRT-PCR Starter Kit (C10712-1, RiboBio, China). The forward primer for hsa-miR-21-5p (miRA0000076-1-200, RiboBio) and the U6 forward primer (miRAN0002-1-200, RiboBio) were used following the manufacturer’s protocol. Quantitative PCR was carried out using an ABIPRISM 7300 Sequence Detection System (Applied Biosystems, USA).

HG-HUVECs and HG-HDFs were treated with NC mimic (HY-R04602, MCE, USA), sEVs^Hypo-5%^ + inhibitor^NC^ (HY-RI04602, MCE, USA), sEVs^Hypo-5%^ + inhibitor^miR-21-5p^ (HY-RI00449, MCE), or miR-21-5p mimic (HY-R00449, MCE). Total RNA was extracted using the same RNA extraction kit (YI SHAN Biotechnology, China). Reverse transcription and quantitative PCR were performed using the miDETECT A Track miRNA qRT-PCR Starter Kit (C10712-1, RiboBio). Quantitative PCR was conducted using the ABIPRISM 7300 Sequence Detection System.

### Immunofluorescence staining

To assess HG-HDFs activation and phenotypic transition, α-SMA was used as a myofibroblast marker. HG-HDFs at approximately 70% to 80% confluence was fixed with 4% paraformaldehyde for 15 min, permeabilized with 0.3% Triton X-100 for 10 min, and subsequently blocked with 1% bovine serum albumin for 1 h. Cells were incubated with anti-α-SMA (14395-1-AP, Proteintech, China) at 4 °C overnight and then incubated with Alexa Fluor 488-conjugated secondary antibody (A-11008, Invitrogen, USA) for 1 h, followed by Hoechst 33342 for nuclear counterstaining. Images were acquired using a CLSM (Leica, Germany).

For the immunofluorescence staining of the wound tissue, the sections were first deparaffinized in xylene and then rehydrated using a series of ethanol concentrations. Antigen retrieval was performed using a citrate buffer. Next, the sections were permeabilized with 0.3% Triton X-100 and blocked with an immunofluorescence staining blocking solution at room temperature for 1 h. Then, the sections were incubated overnight with antibodies against PCNA (60097-1-Ig, Proteintech, China), CD31 (A19034, Abclonal, China), or α-SMA (14395-1-AP, Proteintech). After washing the sections with PBS, they were incubated with appropriate Alexa Fluor-conjugated secondary antibodies (Invitrogen, USA) at room temperature for 1 h. The nuclei were stained with 4′,6-diamidino-2-phenylindole (DAPI).

### Fabrication and characterization of sEVs^Hypo-5%^-loaded nanoscaffold

Electrospinning solutions were prepared by dissolving 1.5 g of PCL pellets (24980-41-4, Aladdin, China) in a 1:1 (v/v) mixture of tetrahydrofuran (1099-99-9, MACKLIN, China) and *N*,*N*-dimethylformamide (68-12-2, MACKLIN). The mixture was magnetically stirred at room temperature for more than 6 h until a clear and homogeneous solution was obtained. Electrospinning was performed using a precision electrospinning apparatus (ISPLab01, Dk infusetek, China) equipped with a 22-gauge blunt-ended stainless-steel needle. The flow rate was maintained at 21 μl/min, with an applied voltage of 14 to 19 kV and a needle-to-collector distance of 21 cm. The process was conducted under controlled environmental conditions, with the temperature maintained above 30 °C and relative humidity kept below 30%, to ensure stable fiber formation. Electrospinning was carried out for 3 h, after which the collected nanofiber membranes were placed in a vacuum drying chamber to remove any residual solvent.

To enhance the surface bioactivity of the pristine nanoscaffold and facilitate subsequent biomolecule immobilization, PDA coating was applied via DA self-polymerization. Pristine nanoscaffolds were presoaked in anhydrous ethanol and then incubated overnight in a DA hydrochloride solution (2 mg/ml in Tris-HCl buffer, 10 mM, pH 8.5) under constant agitation at room temperature. The PDA-coated nanoscaffold served as a reactive platform for subsequent surface functionalization and biomolecule immobilization. Finally, the scaffolds were ultrasonically washed several times with deionized water until the rinse solution remained clear, and then dried in a vacuum oven. The morphology of the scaffolds was examined using SEM (ZEISS Sigma 360, Germany). Mechanical properties were evaluated using a universal testing machine (MARK-10, USA). Rectangular specimens (10 mm × 50 mm) were subjected to uniaxial tensile testing at a crosshead speed of 10 mm/min. The static water contact angle was measured using a goniometer (KRUSS-DSA100**,** Germany).

To quantitatively evaluate the adsorption and loading capacity of sEVs on the scaffolds, the pristine nanoscaffold or the PDA-coated nanoscaffold (diameter: 10 mm) was incubated with sEVs at varying concentrations (10 to 50 μg/ml) at 4 °C for 30 min. Following incubation, the scaffolds were washed 3 times with sterile PBS to remove unbound sEVs, and the resulting supernatants were collected for quantification. The residual protein content in the collected supernatants was quantified using a BCA protein assay kit (PC0020, Solarbio). Loading efficiency was calculated as the percentage of sEVs protein adsorbed onto the scaffolds relative to the total initial input.

To evaluate the sustained release behavior of sEVs, the sEVs^Hypo-5%^-loaded nanoscaffold and the PDA-coated nanoscaffold were incubated in PBS at 37 °C with gentle agitation. Supernatants were collected at predetermined time points (D1, D2, D3, D5, D7, D10, D12, and D14), and an equal volume of fresh PBS was added back after each sampling. The total protein content of released sEVs in each collected fraction was quantified using a BCA protein assay kit (PC0020, Solarbio), and cumulative release profiles were plotted as cumulative release (%) versus time.

To observe the uptake dynamics of sEVs^Hypo-5%^ continuously released from sEVs^Hypo-5%^-loaded nanoscaffold in HG-HUVECs, sEVs^Hypo-5%^ were first labeled with Dil, and the PDA-coated nanoscaffolds were incubated in the Dil-labeled vesicle suspension to allow vesicle loading. Subsequently, a coculture system was established using a Transwell chamber with 8-μm pores. HG-HUVECs (4 × 10^4^ cells/well) were seeded in the lower chamber while the sEVs^Hypo-5%^-loaded nanoscaffolds were placed in the upper chamber; free sEVs^Hypo-5%^ served as the control. After coculture for 12, 24, 48, and 72 h, the cytoskeleton was stained with phalloidin (YESEN Biotechnology, Shanghai, China), and nuclei were counterstained with DAPI. The intracellular distribution and uptake dynamics of sEVs were visualized using a fluorescence microscope.

To evaluate the in vivo retention of sEVs^Hypo-5%^ continuously released from the sEVs^Hypo-5%^-loaded nanoscaffold, 2 treatment groups were established. In the control group, 100 μl of Dil-labeled sEVs^Hypo-5%^ was injected subcutaneously at 4 points around the wound margins. In contrast, wounds in the experimental group were covered with the sEVs^Hypo-5%^-loaded nanoscaffold. Fluorescence signals from Dil-labeled sEVs^Hypo-5%^ were monitored using an in vivo imaging system (IVIS, USA) on D1, D3, D7, D10, and D12 post-operations to assess sEVs retention and release dynamics at the wound site.

### Hemolysis assay

Hemolysis test was performed to examine the blood compatibility of the sEVs^Hypo-5%^-loaded nanoscaffold. Fresh whole blood was collected and anticoagulated. Red blood cells (RBCs) were obtained by centrifugation and washed with PBS. RBC suspension was incubated with ddH₂O (positive control), PBS (negative control), free sEVs^Hypo-5%^, hydrocolloid dressing extract, pristine nanoscaffold extract, or sEVs^Hypo-5%^-loaded nanoscaffold extract at 37 °C for 2 h. Samples were then centrifuged at 3,000 rpm for 10 min. The supernatant was collected and measured at 540 nm using a microplate spectrophotometer. Hemolysis ratio (%) was calculated based on the absorbance values.

### Live/dead staining

Live/Dead staining was used to check cell viability after different treatments. HG-HUVECs and HG-HDFs were treated with PBS (Control), free sEVs^Hypo-5%^, hydrocolloid dressing, pristine nanoscaffold, or sEVs^Hypo-5%^-loaded nanoscaffold. After 24 h, cells were stained with Calcein-AM/propidium iodide. Images were taken under a fluorescence microscope. Live cells were stained green and dead cells were stained red.

### Serum biochemical analysis

Blood samples were collected from diabetic mice on postoperative day 14 (D14). Serum was isolated by centrifugation and used for biochemical analysis, including ALT, AST, urea, CREA, and CK-MB.

### Biocompatibility of the scaffolds

For in vivo biocompatibility evaluation, hydrocolloid dressing, pristine nanoscaffold, and sEVs^Hypo-5%^-loaded nanoscaffold were implanted into full-thickness skin defect models in mice. Animals were euthanized on postoperative D7 and D14, and major organs (heart, liver, spleen, lung, and kidney) were collected. The tissues were fixed in 4% paraformaldehyde for 24 h, dehydrated in graded ethanol, embedded in paraffin, sectioned, and subjected to H&E staining to assess possible systemic toxicity.

### Diabetic wound model and treatment protocol

Male BKS-DBDB mice (SPF, 7W, VIEWSOLID, China) were used to establish a chronic diabetic wound model. Mice were anesthetized by intraperitoneal injection of 1% pentobarbital sodium (0.3 ml/30 g), and 2 full-thickness excisional wounds (10 mm in diameter) were created symmetrically on the dorsal skin while carefully avoiding injury to the underlying muscle.

Mice were randomly assigned to 5 experimental groups: (a) control group; (b) free sEVs^Hypo-5%^ group; (c) hydrocolloid group: wounds were treated with a commercial hydrocolloid dressing; (d) pristine nanoscaffold group: wounds treated with PCL without sEVs^Hypo-5%^ loading; and (e) sEVs^Hypo-5%^-loaded nanoscaffold group: wounds treated with PCL scaffolds loaded with sEVs^Hypo-5%^.

Wound healing progression was recorded by digital photography on postoperative D0, D3, D7, D10, and D14. The wound closure rate was calculated using the following formula: Wound closure rate (%) = (Initial wound area – Current wound area) / Initial wound area × 100%.

Local blood perfusion was assessed on postoperative D7 and D14 using laser Doppler perfusion imaging (LDPI, PeriMed PeriScan, COSLAN, China). Subsequently, wound tissues and peri-wound skin were harvested for histopathological and immunofluorescence analyses.

### Statistical analysis

Data are presented as mean ± SD. Analyses were performed in GraphPad Prism 8.0. Two-group comparisons used *t* tests; comparisons among more than 2 groups used one-way analysis of variance. *P* < 0.05 was considered statistically significant.

## Ethical Approval

All animal protocols were approved by the Institutional Animal Care and Use Committee of Beijing Viewsolid Biotechnology Co. Ltd. (approval numberVS2126A00741).

## Data Availability

The data that support the findings of this study are available from the corresponding authors upon reasonable request.
