# Supplementary material for: Hypoxia-Challenged sEVs-Engineered Nanofiber Scaffolds Accelerate Diabetic Wound Healing via Reversing Cellular Dysfunction of Skin Repair Cells
Source: Research (Wash D C). 2026 May 7;9:1248. doi: 10.34133/research.1248 (PMC13150076; doi:10.34133/research.1248)
Supplement: Supplementary 1 — Figs. S1 to S15 [file research.1248.f1.zip › renamed_733fb.docx]

**Hypoxia-Challenged sEVs Engineered Nanofiber Scaffolds Accelerate Diabetic Wound Healing via Reversing Cellular Dysfunction of Skin Repair Cells**


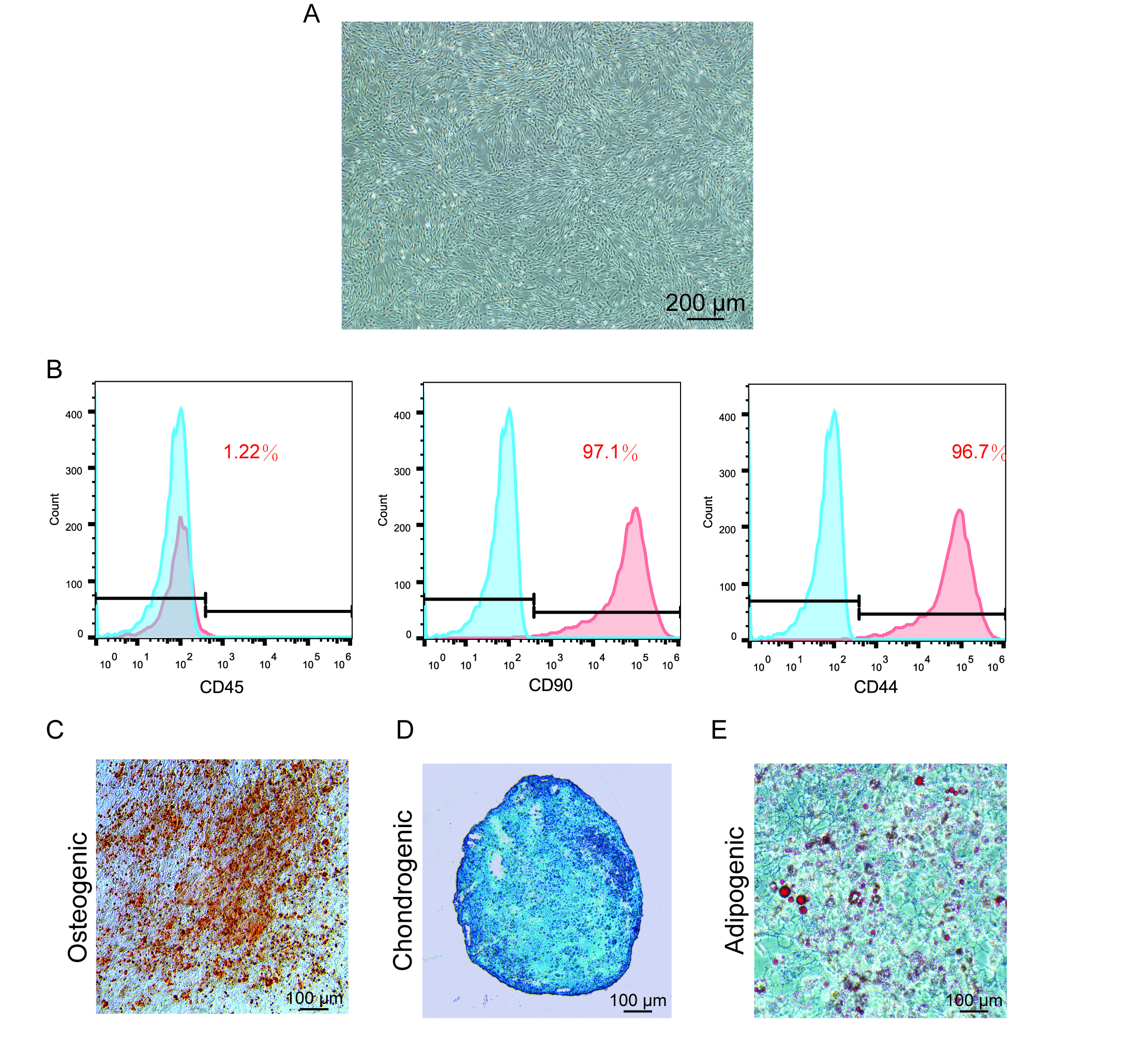


**Fig. S1**. Characterization of CP-MSCs. (A, B) Representative images showing the morphology of CP-MSCs at different density. Scale bar = 200 μm. (C) Flow cytometry analysis of surface marker expression in CP-MSCs, including CD45, CD90, and CD44. (D) Alizarin Red staining for osteogenic differentiation. Scale bar = 100 μm. (E) Alcian Blue staining for chondrogenic differentiation. Scale bar = 100 μm. (F) Oil Red O staining for adipogenic differentiation. Scale bar = 100 μm.


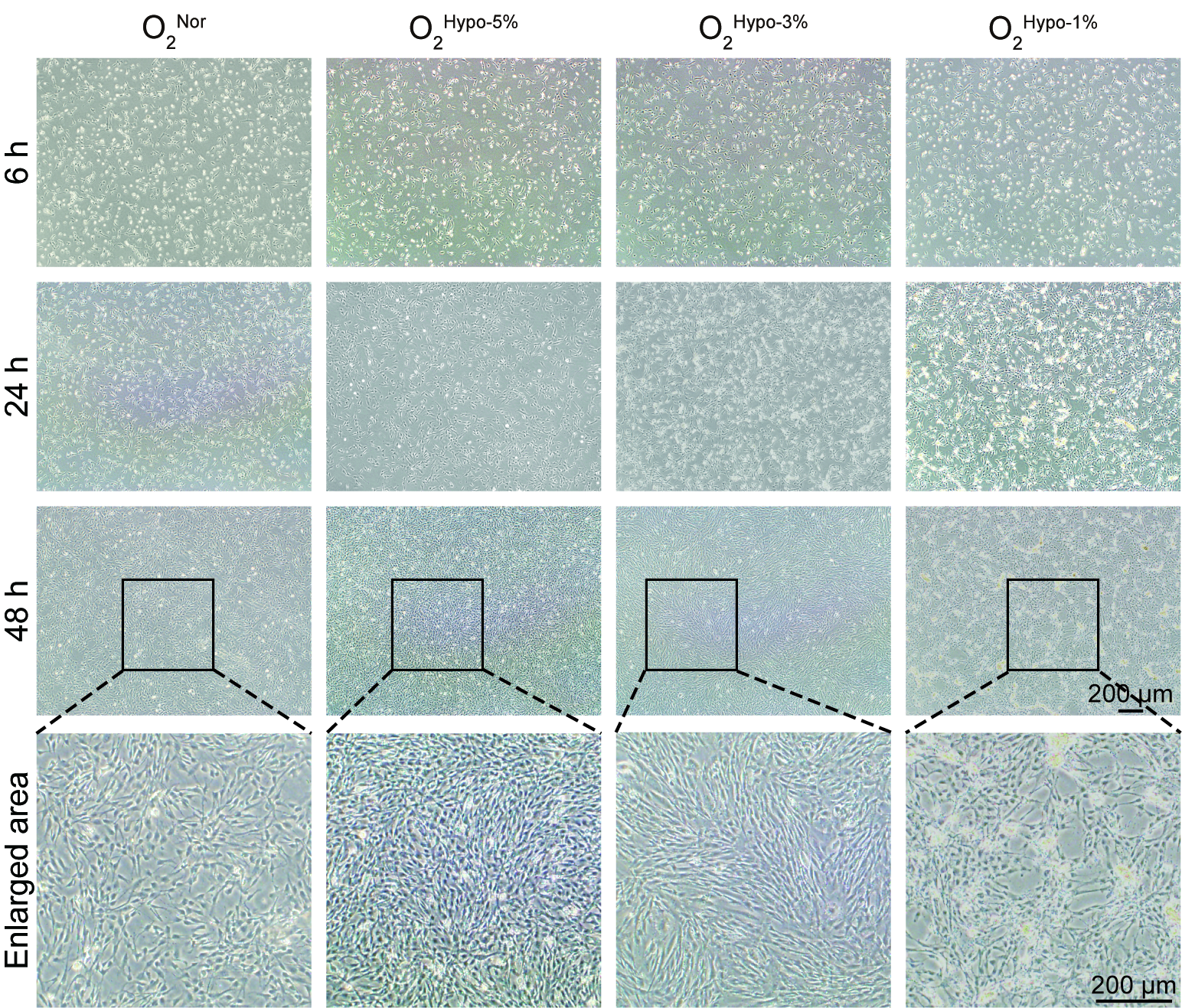


**Fig. S2.** Representative images of CP-MSCs cultured under normoxia (O₂^Nor^), 5%O₂ (O₂^Hypo-5%^), 3%O₂ (O₂^Hypo-3%^), and 1%O₂ (O₂^Hypo-1%^). Scale bar = 200 μm.


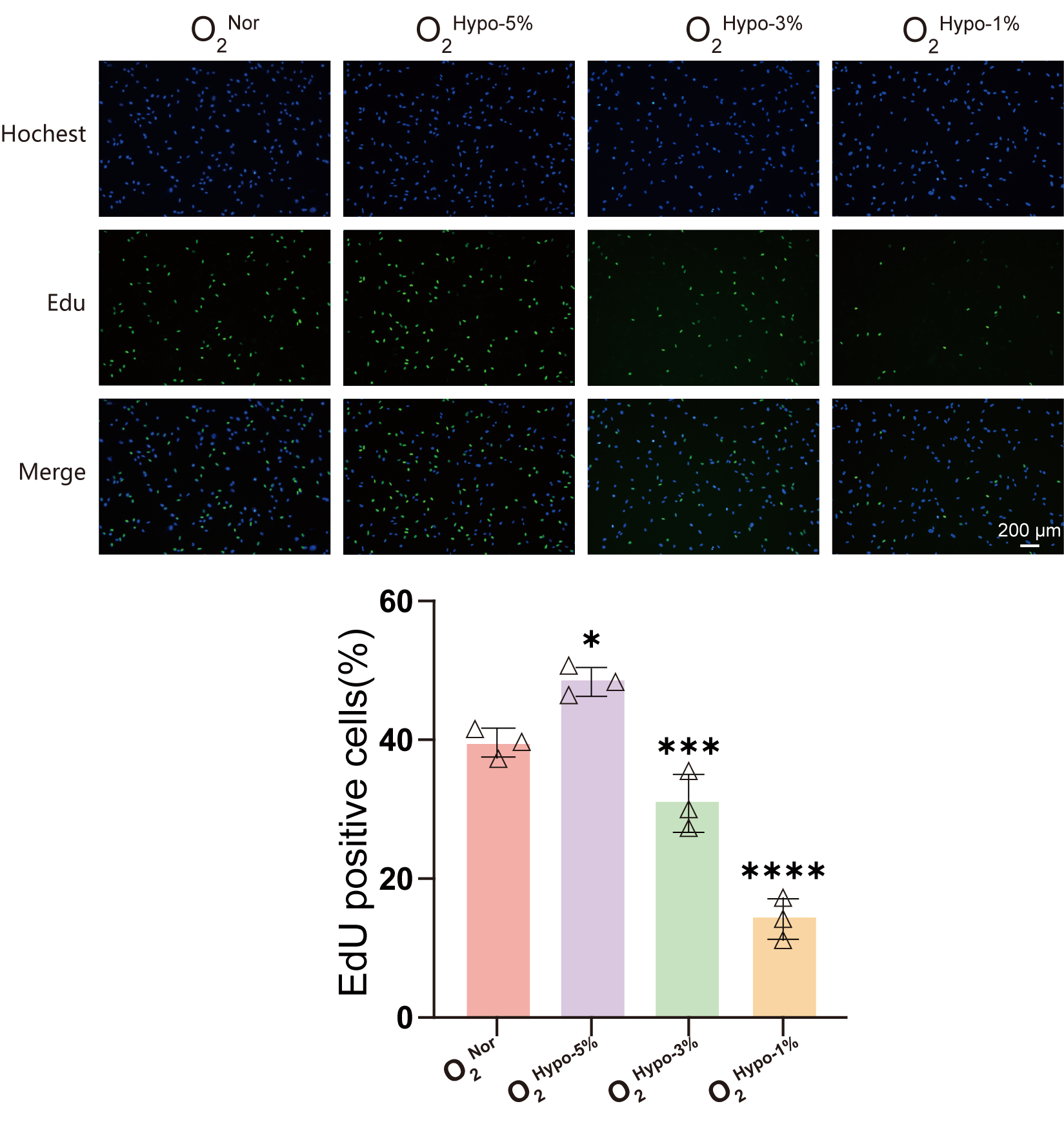


**Fig. S3.**  EdU staining of proliferating CP-MSCs under each oxygen condition, scale bar =200 μm. (C) Quantification of EdU⁺ cells. ns, not significant, **P* < 0.05, ***P* < 0.01, ****P* < 0.001, *****P* < 0.0001. n = 3 per group.


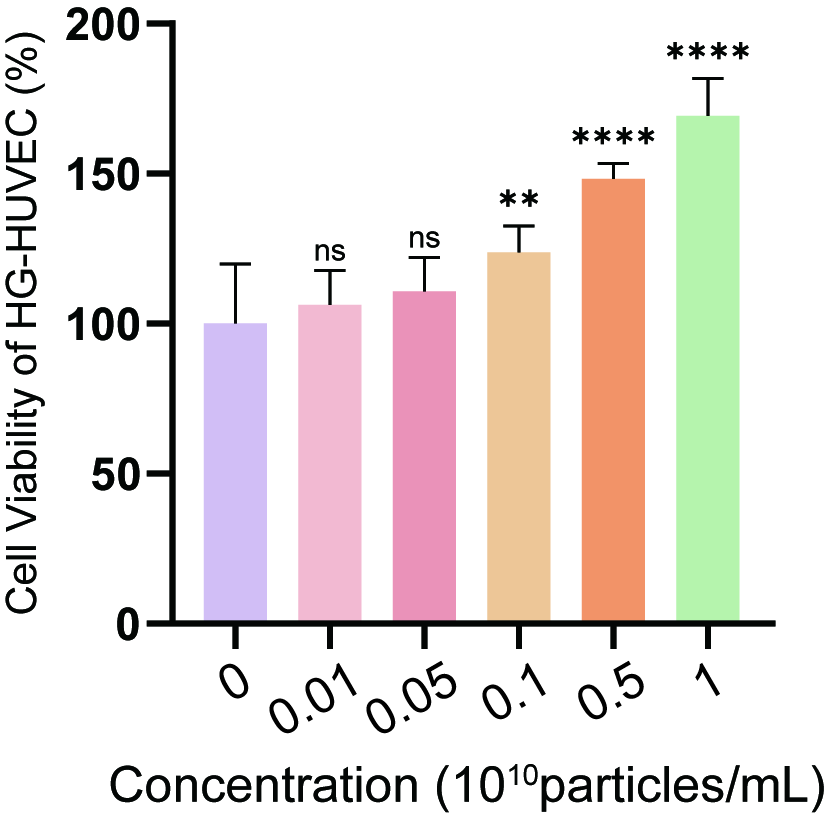


**Fig. S4.** Cell viability of HG-HUVEC after treatment with different concentrations of sEVs (0–1×10¹⁰ particles/mL) for 24 h, as determined by the CCK-8 assay. ns, not significant, **P* < 0.05, ***P* < 0.01, ****P* < 0.001, *****P* < 0.0001. n = 5 per group.


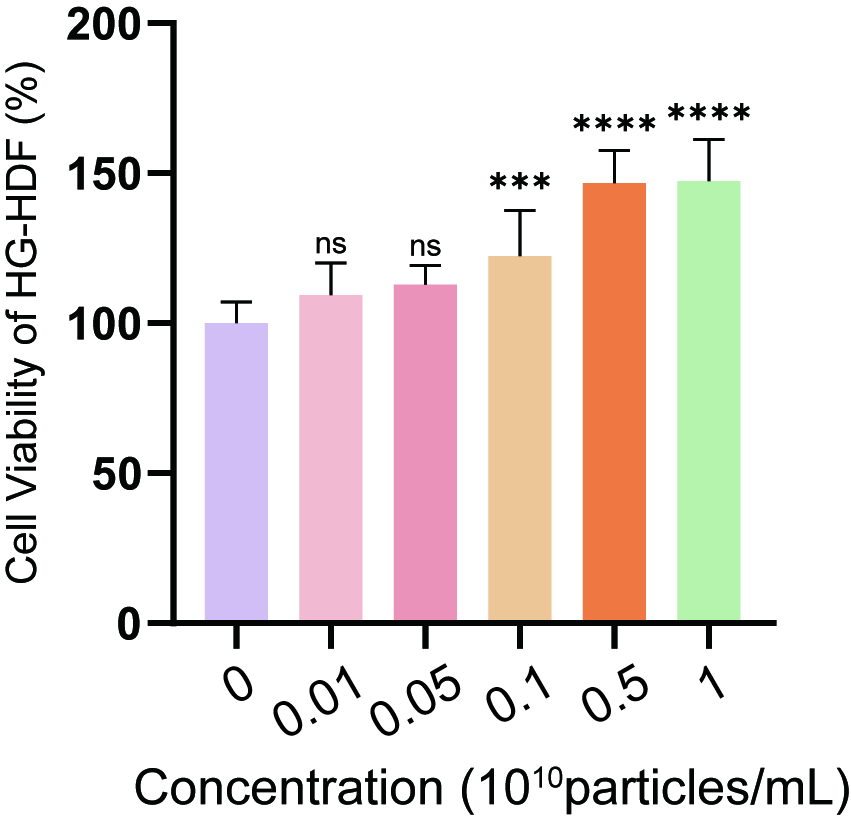


**Fig. S5.** Cell viability of HG-HDF after treatment with different concentrations of sEVs (0-1×10¹⁰ particles/mL) for 24 h, as determined by the CCK-8 assay. ns, not significant, **P* < 0.05, ***P* < 0.01, ****P* < 0.001, *****P* < 0.0001. n = 5 per group.


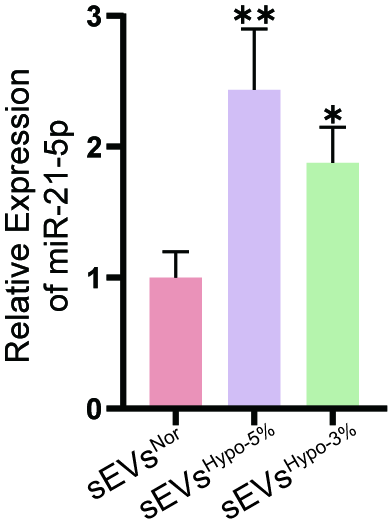


**Fig. S6. Relative expression of miR-21-5p in sEVs derived from CP-MSCs under different oxygen conditions.** qRT-PCR analysis of miR-21-5p expression in sEVs^Nor^, sEVs^Hypo-5%^, sEVs^Hypo-3%^. ns, not significant, **P* < 0.05, ***P* < 0.01, ****P* < 0.001, *****P* < 0.0001. n = 3 per group.


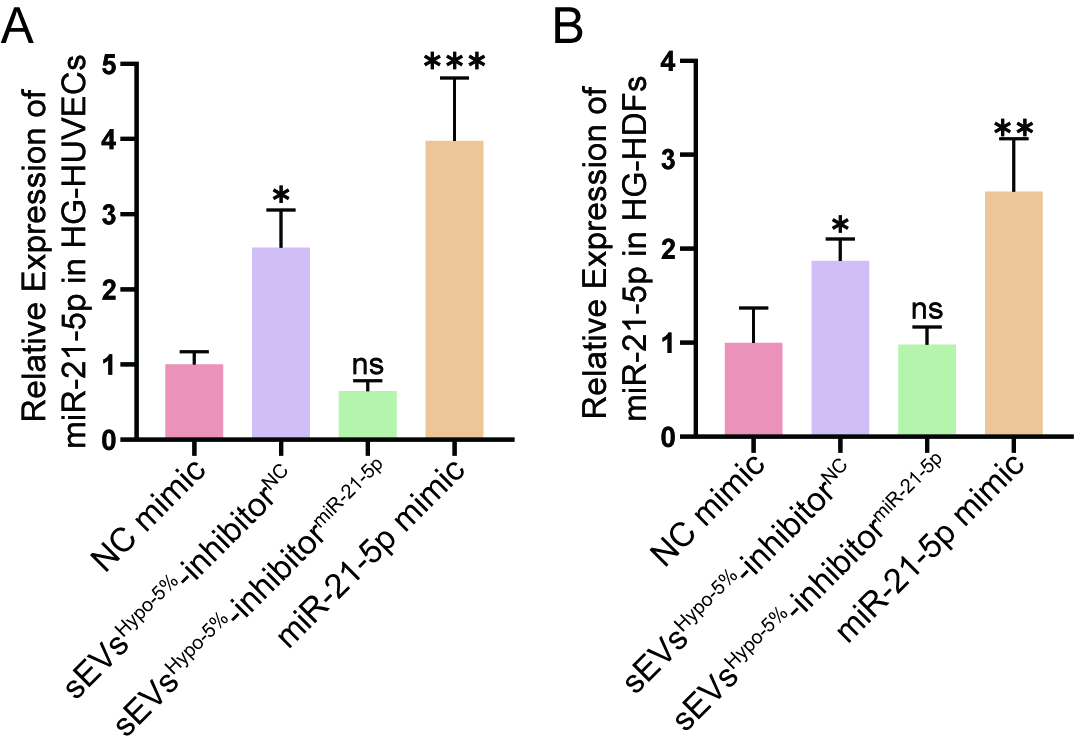


**Fig. S7. MiR-21-5p expression in HG-HUVECs (A) and HG-HDFs (B)** treated with NC mimic, sEVs^Hypo-5%^ + inhibitor^NC^, sEVs^Hypo-5%^ + inhibitor^miR-21-5p^, or miR-21-5p mimic**.**ns, not significant, **P* < 0.05, ***P* < 0.01, ****P* < 0.001, *****P* < 0.0001. n = 3 per group.


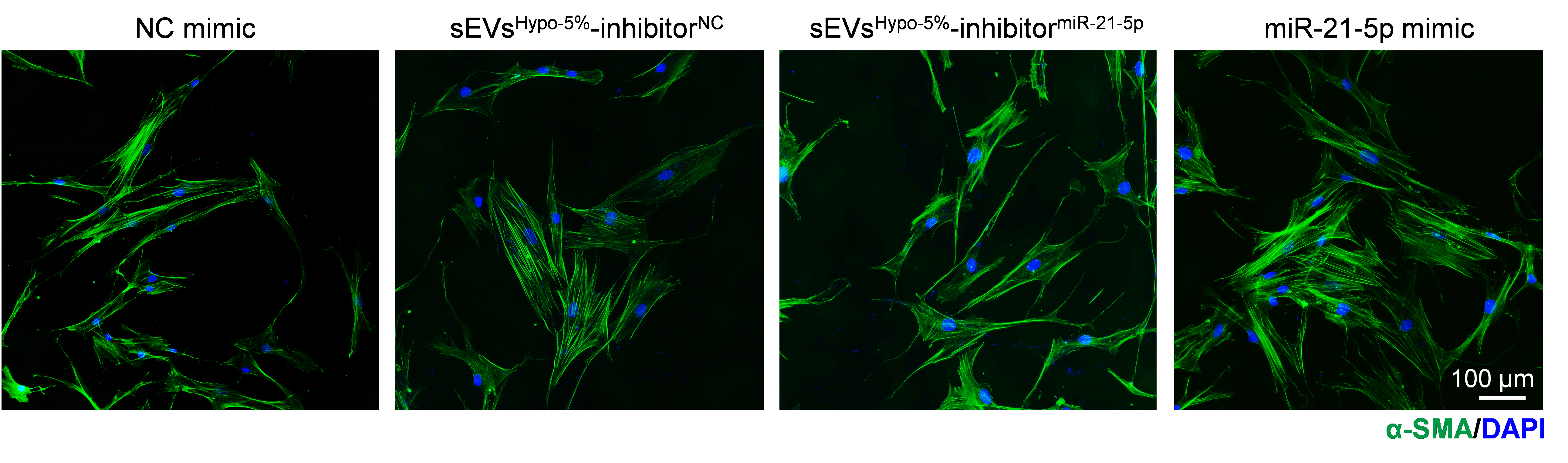


**Fig. S8. Effect of miR-21-5p on α-SMA expression in HG-HDFs.** Representative immunofluorescence images of α-SMA expression in HG-HDFs under different treatments. α-SMA is shown in green and nuclei are stained with DAPI (blue). Scale bar = 100 μm.


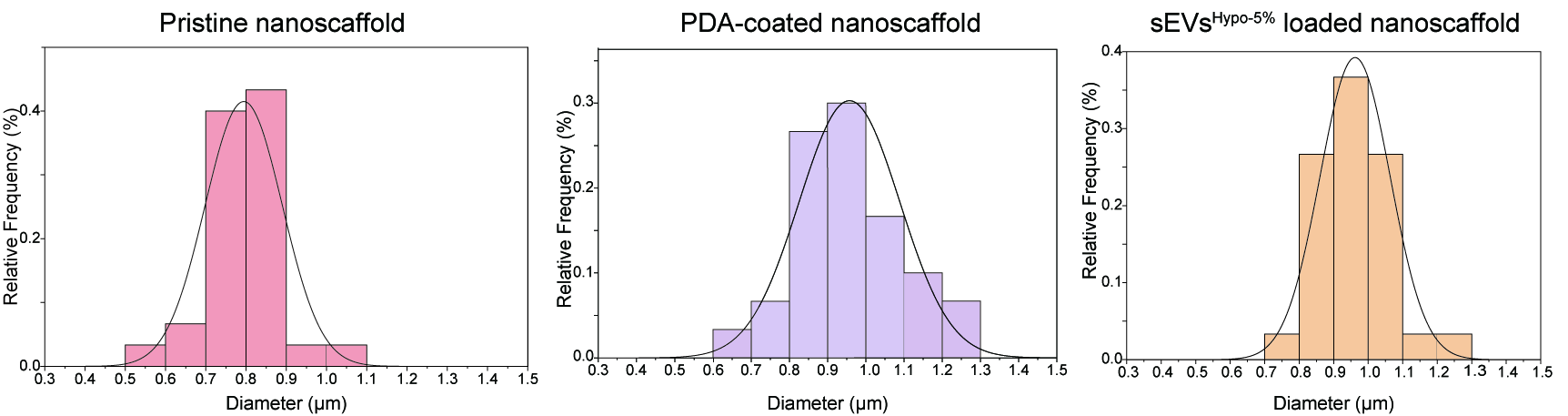


**Fig. S9.** Fiber diameter distribution of different scaffolds. Histograms showing the diameter distribution of electrospun fibers in Pristine nanoscaffold, PDA-coated nanoscaffold, and sEVs^Hypo-5%^ loaded nanoscaffold.


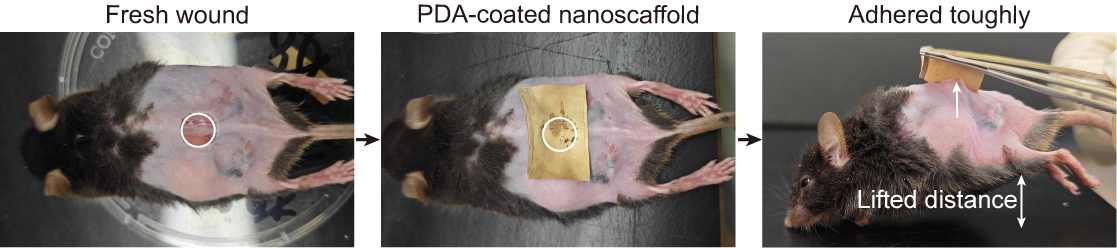


**Fig. S10.** Representative photographs showing the wet adhesion of the PDA-coated nanoscaffold on the fresh wound.


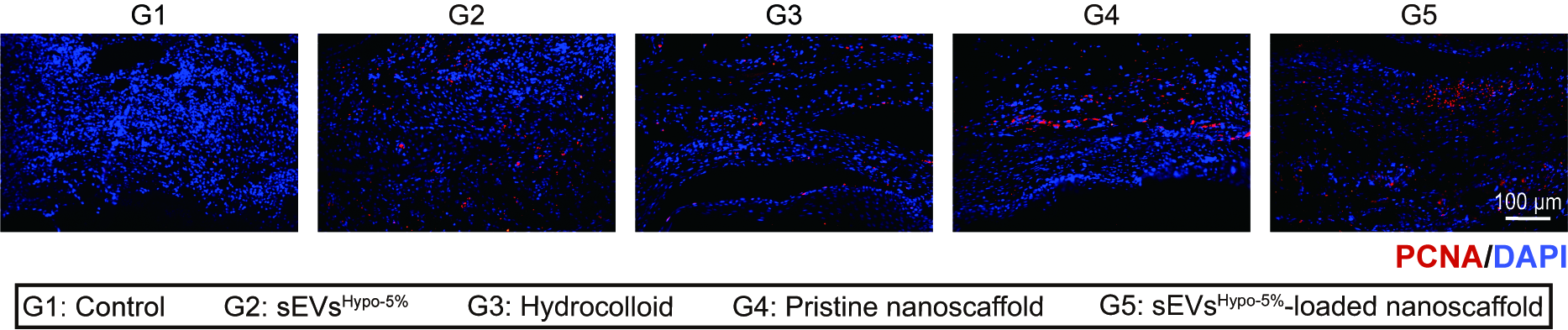


**Fig. S11. PCNA expression in wound tissue.** Representative immunofluorescence images of PCNA staining in wound sections. PCNA-positive nuclei are shown in red, and nuclei are stained with DAPI (blue). Scale bar = 100 μm.


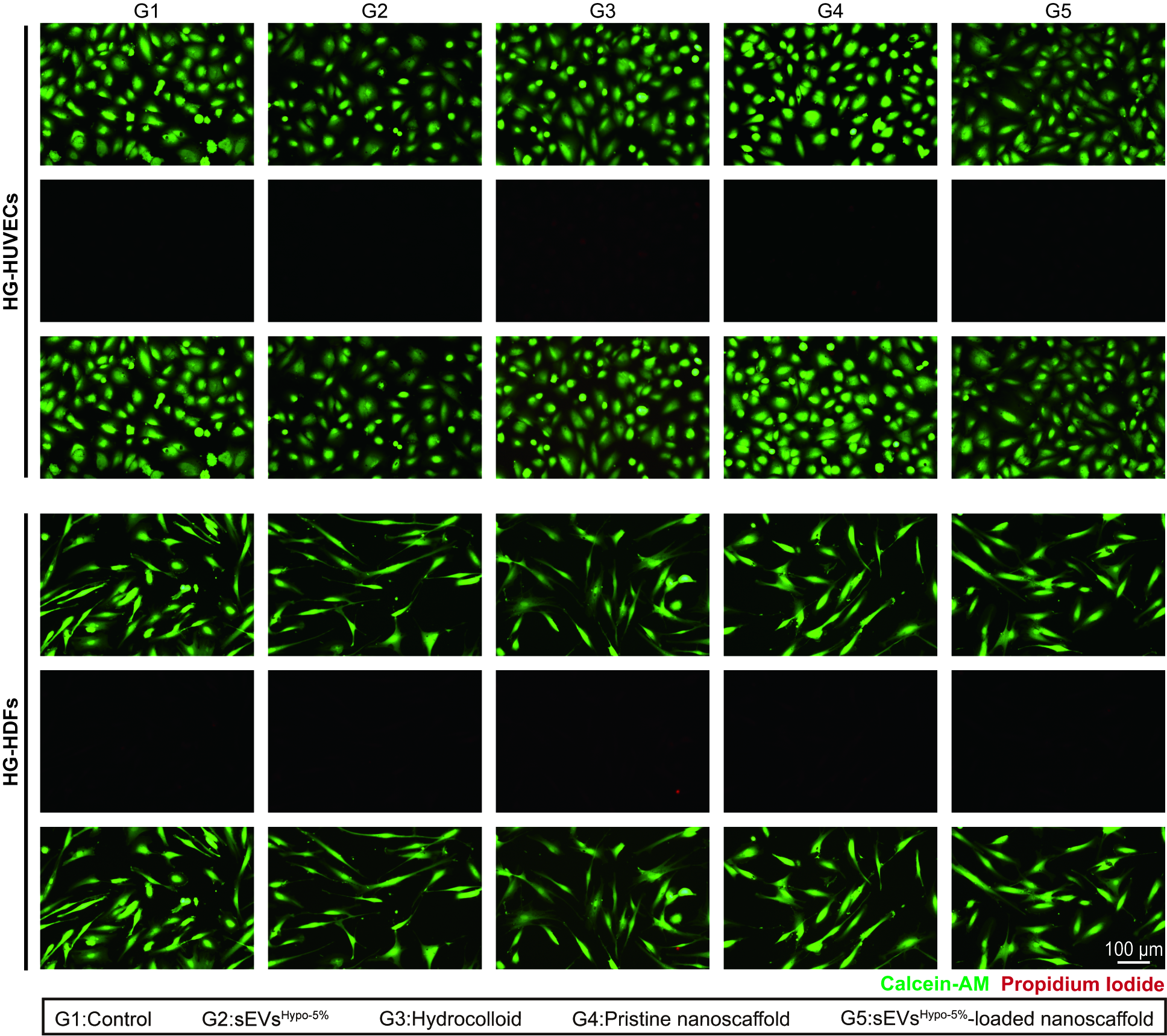


**Fig. S12.** Representative Live/Dead fluorescence images of HG-HUVECs and HG-HDFs after treatment with PBS (Control), free sEVs^Hypo-5%^, hydrocolloid dressing, Pristine nanoscaffold, or sEVs^Hypo-5%^-loaded nanoscaffold. Calcein-AM (green) and propidium iodide (red). Scale bar = 100 μm. n = 5 per group.


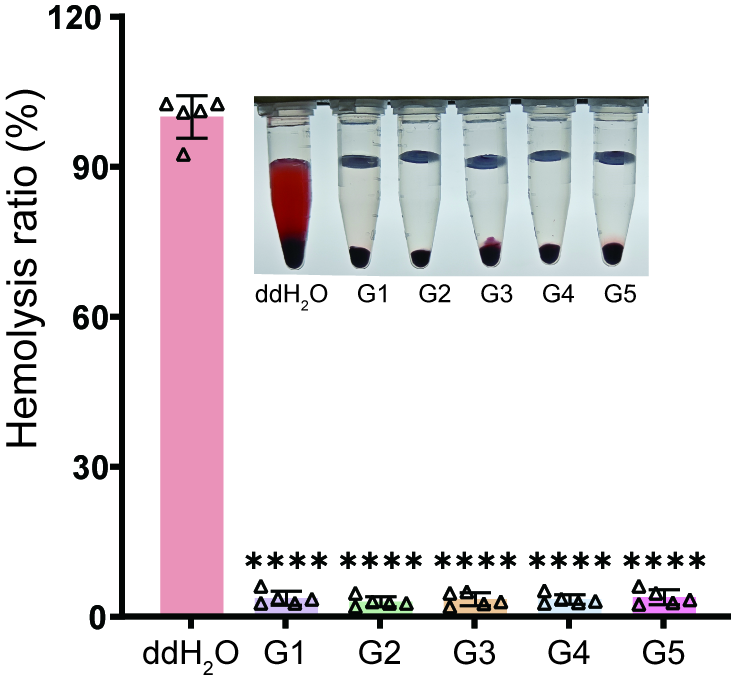


**Fig. S13.** Hemolysis ratio of red blood cells incubated with ddH₂O, Control, free sEVs^Hypo-5%^, hydrocolloid dressing, Pristine nanoscaffold, or sEVs^Hypo-5%^-loaded nanoscaffold. ns, not significant, **P* < 0.05, ***P* < 0.01, ****P* < 0.001, *****P* < 0.0001. n = 5 per group.


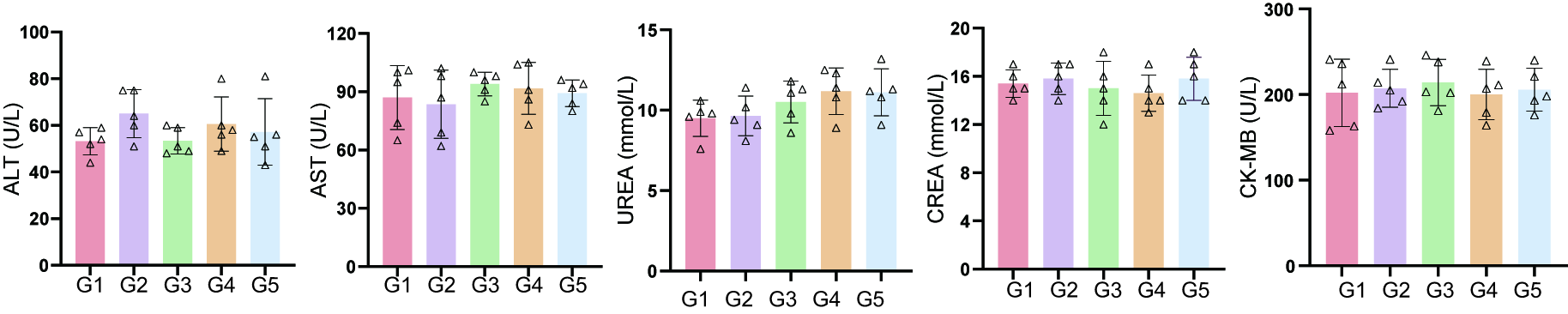


**Fig. S14.** Serum biochemical parameters of diabetic mice in different treatment groups on postoperative day 14 (D14), including ALT, AST, UREA, CREA, and CK-MB. ns, not significant, **P* < 0.05, ***P* < 0.01, ****P* < 0.001, *****P* < 0.0001. n = 5 per group.


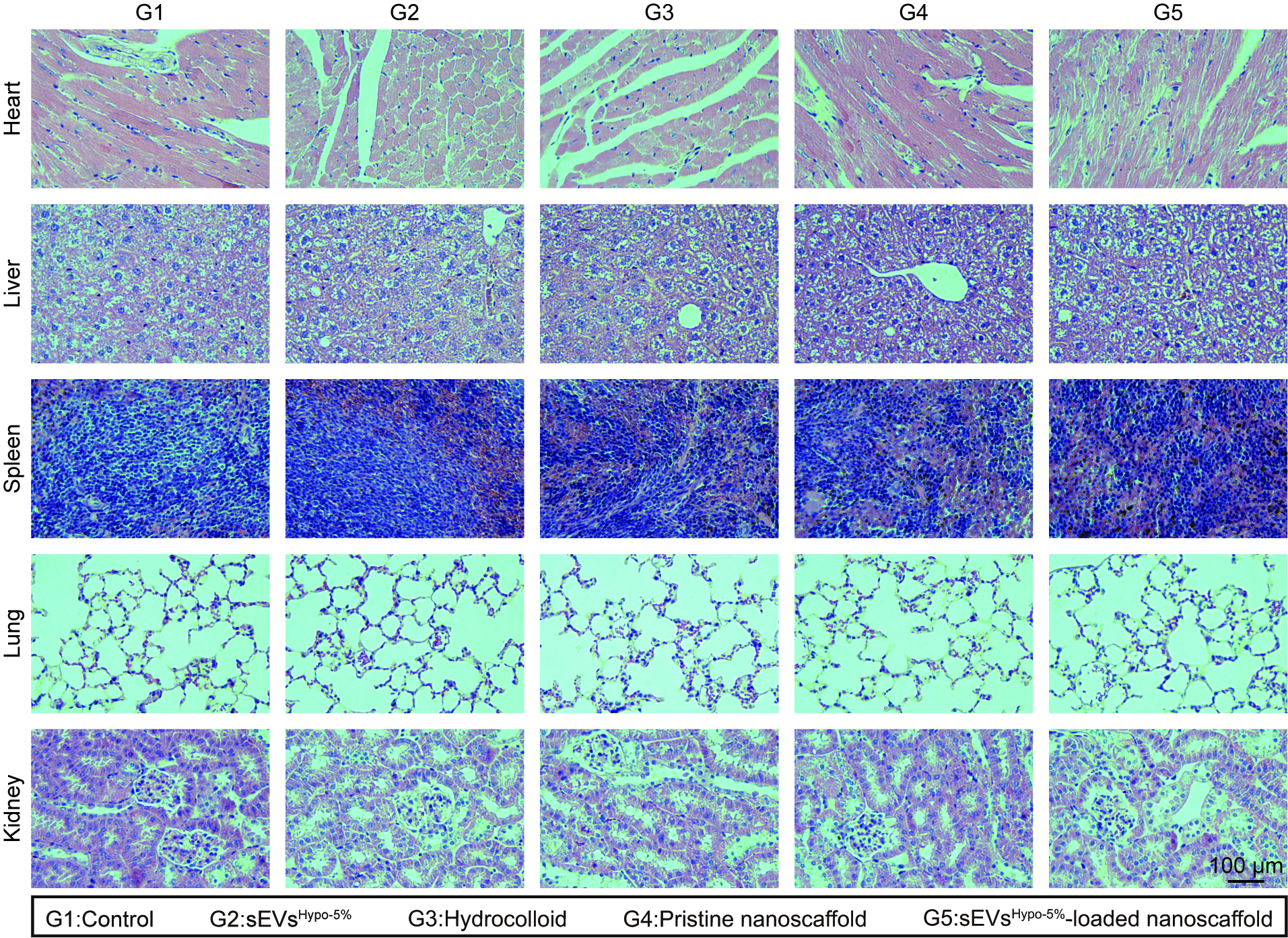
**Fig. S15.** H&E staining of major organs (heart, liver, spleen, lung, and kidney) harvested from diabetic mice in different treatment groups on postoperative day 14 (D14).
