# Supplementary figures and images for: Hypoxia-Challenged sEVs-Engineered Nanofiber Scaffolds Accelerate Diabetic Wound Healing via Reversing Cellular Dysfunction of Skin Repair Cells
Source: Research (Wash D C). 2026 May 7;9:1248. doi: 10.34133/research.1248 (PMC13150076; doi:10.34133/research.1248)

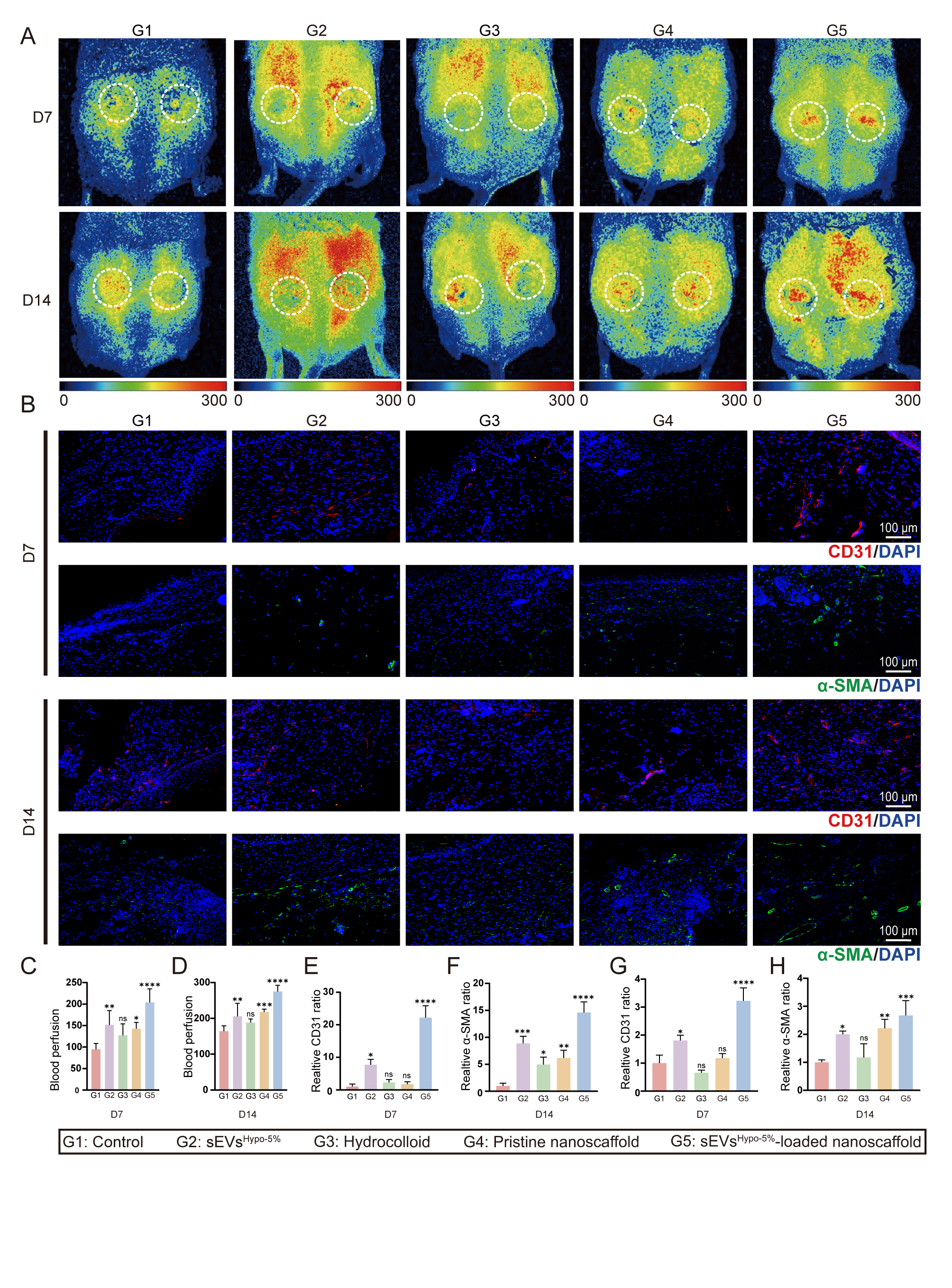

Supplement: Supplementary 1 — Figs. S1 to S15 [file research.1248.f1.zip › Fig 8.tif]

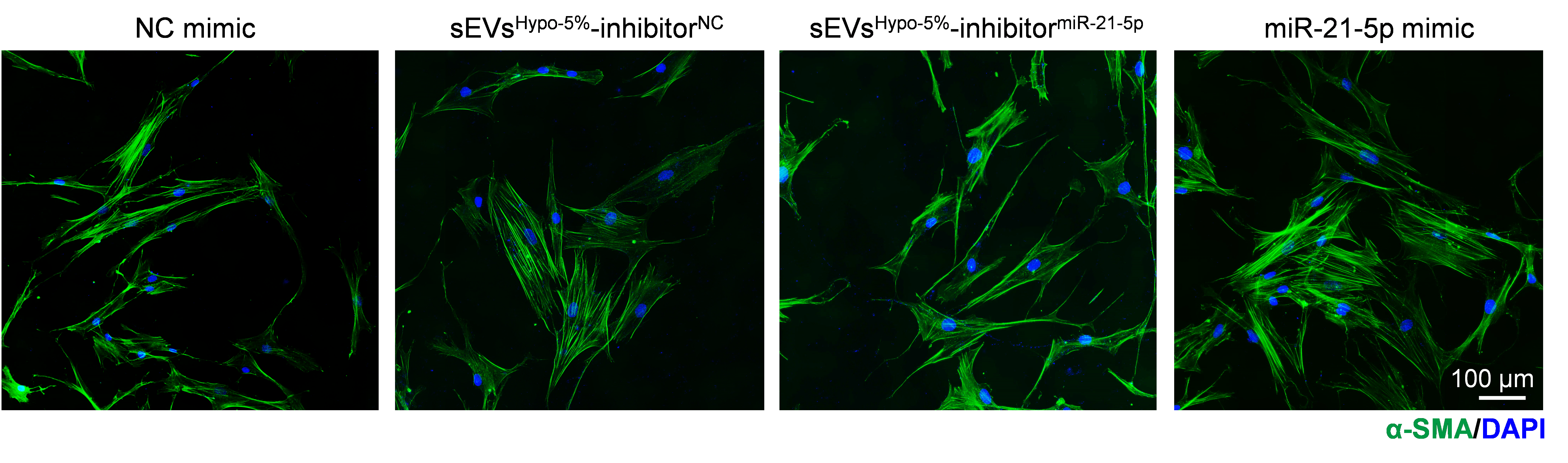

Supplement: Supplementary 1 — Figs. S1 to S15 [file research.1248.f1.zip › Fig S8.tif]

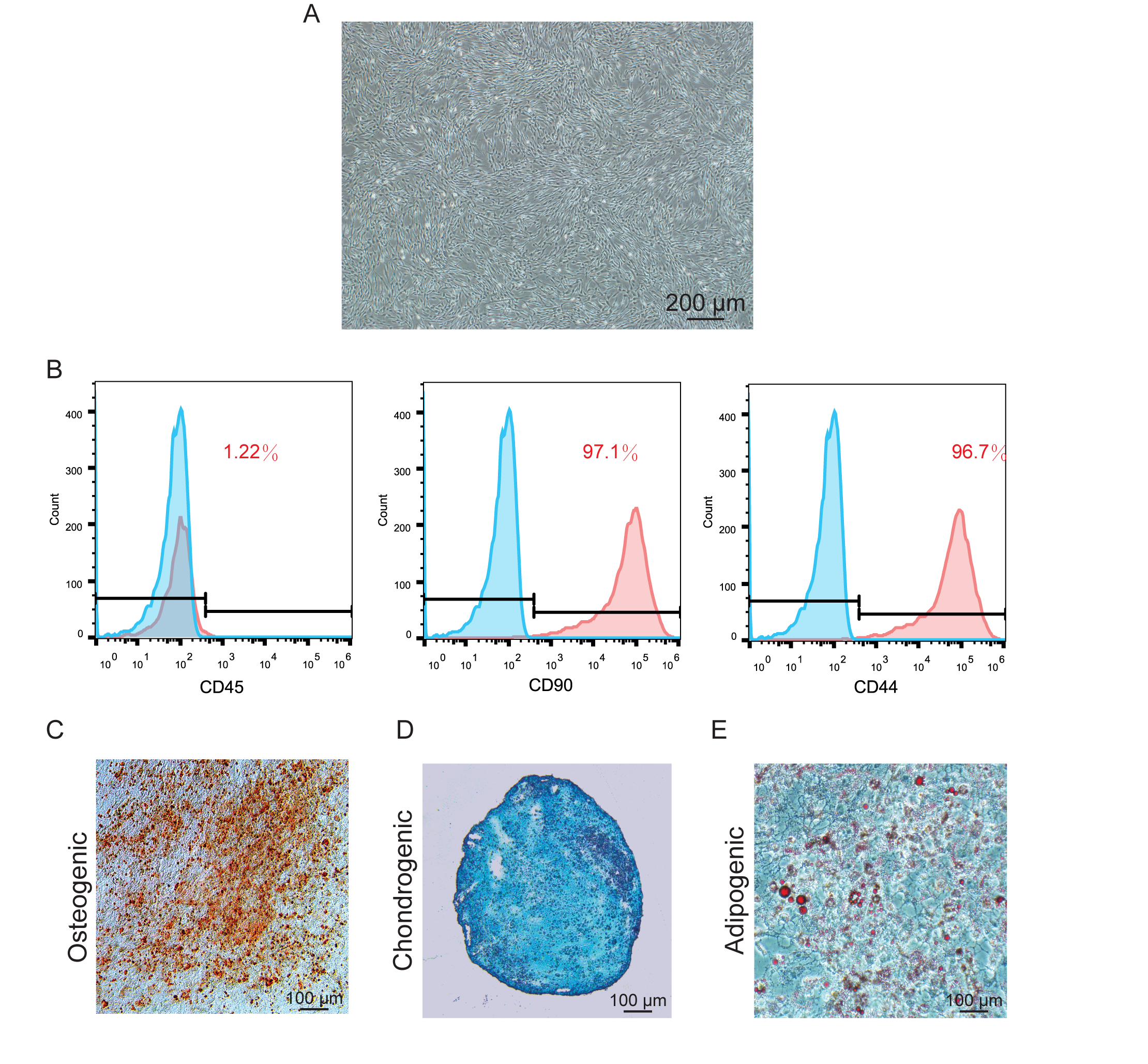

Supplement: Supplementary 1 — Figs. S1 to S15 [file research.1248.f1.zip › Fig.S1.tif]

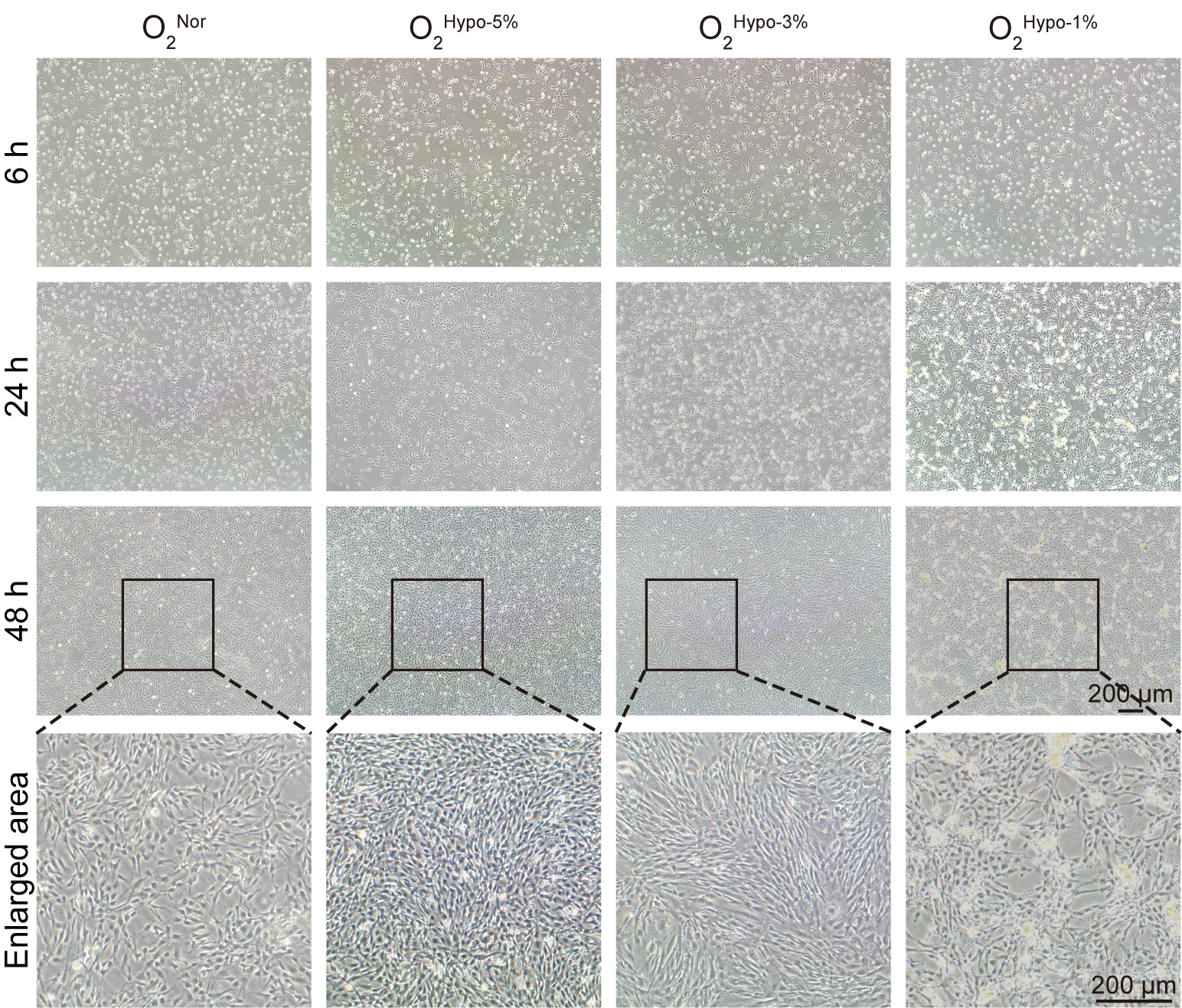

Supplement: Supplementary 1 — Figs. S1 to S15 [file research.1248.f1.zip › Fig.S2.tif]

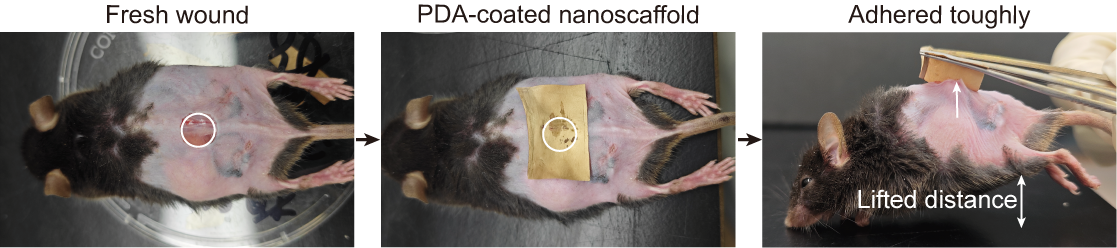

Supplement: Supplementary 1 — Figs. S1 to S15 [file research.1248.f1.zip › S10.tif]

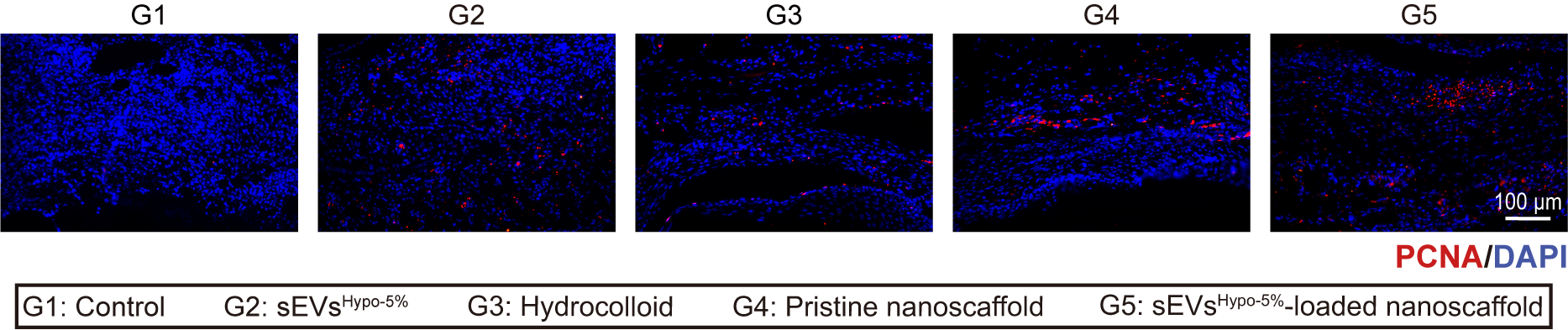

Supplement: Supplementary 1 — Figs. S1 to S15 [file research.1248.f1.zip › S11.tif]

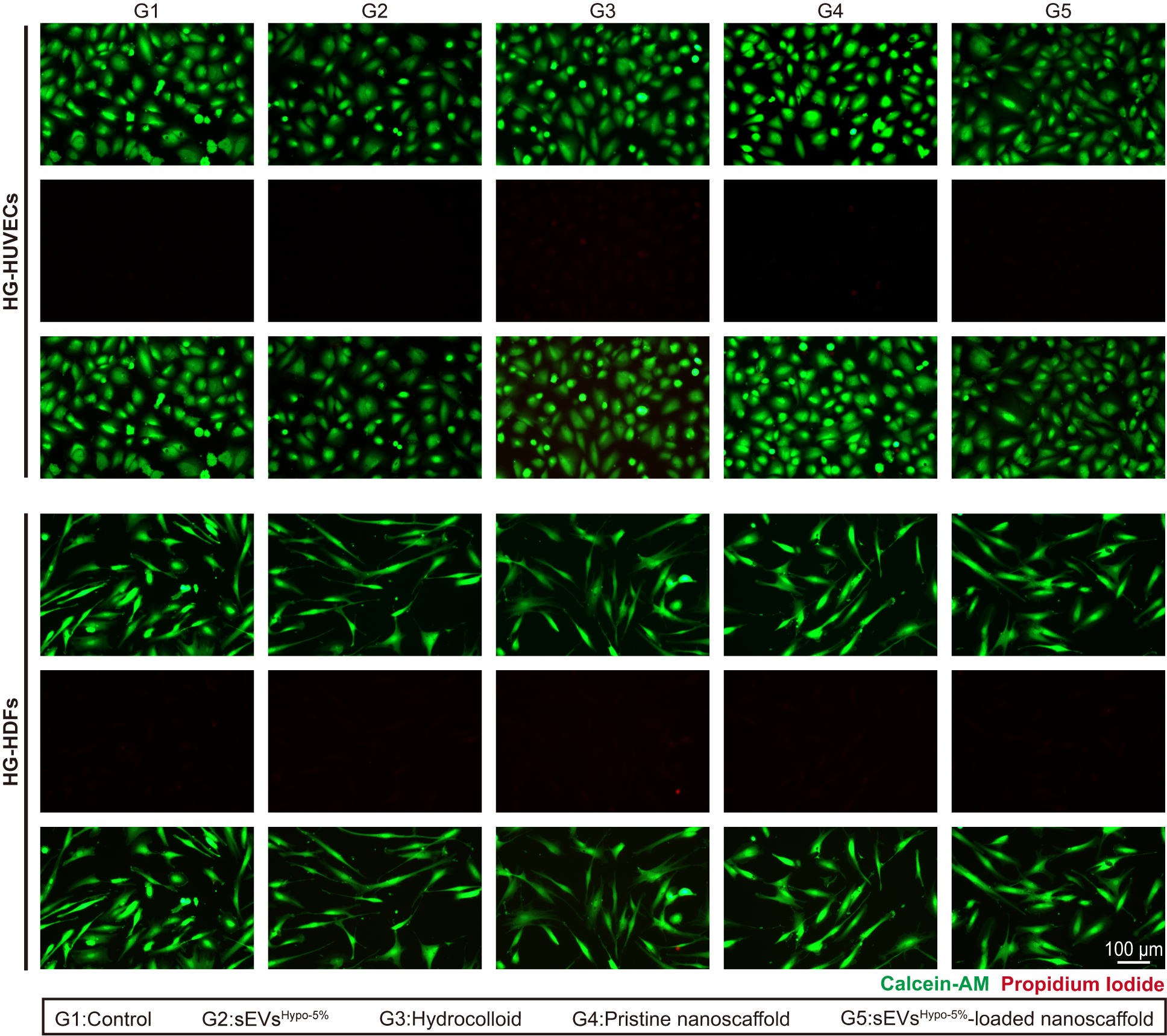

Supplement: Supplementary 1 — Figs. S1 to S15 [file research.1248.f1.zip › S12.tif]

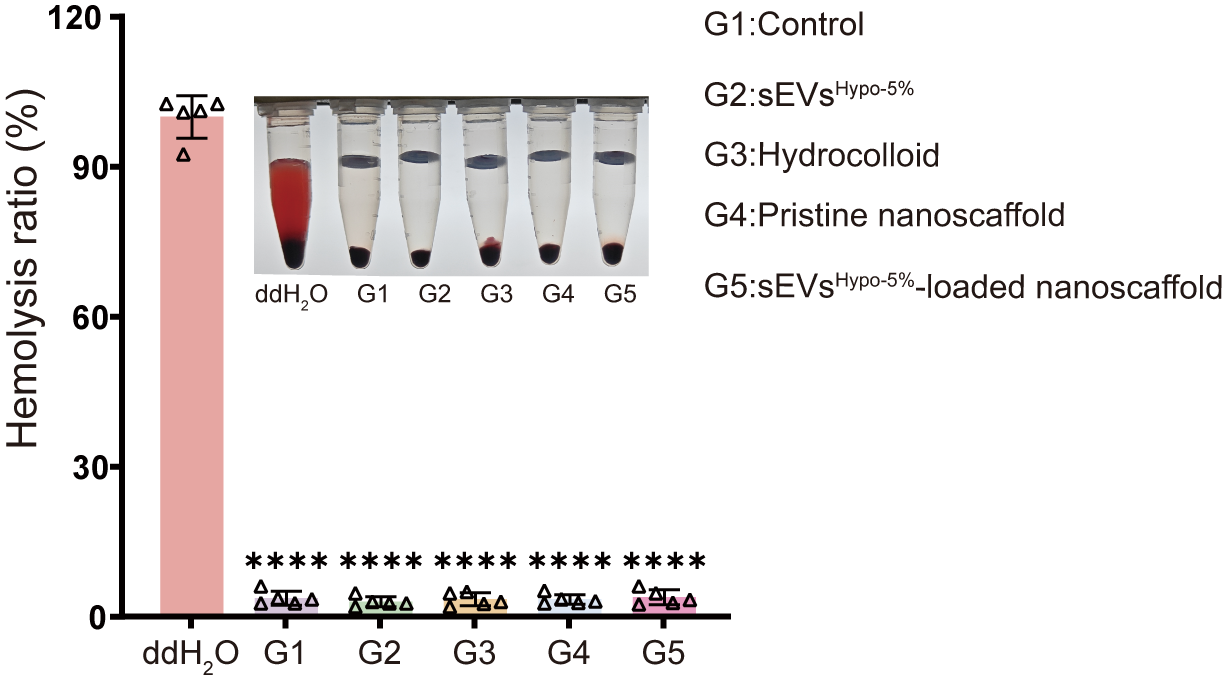

Supplement: Supplementary 1 — Figs. S1 to S15 [file research.1248.f1.zip › S13.tif]

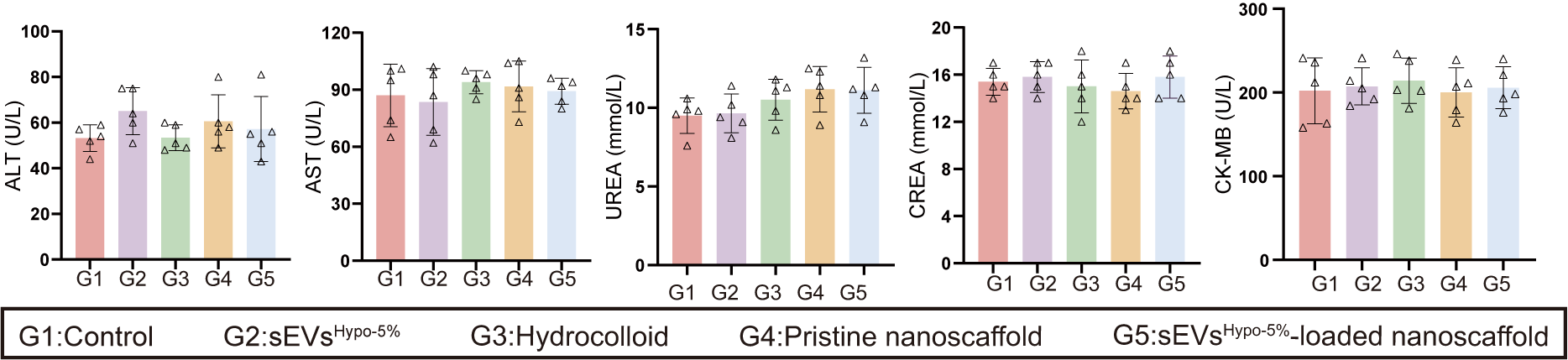

Supplement: Supplementary 1 — Figs. S1 to S15 [file research.1248.f1.zip › S14.tif]

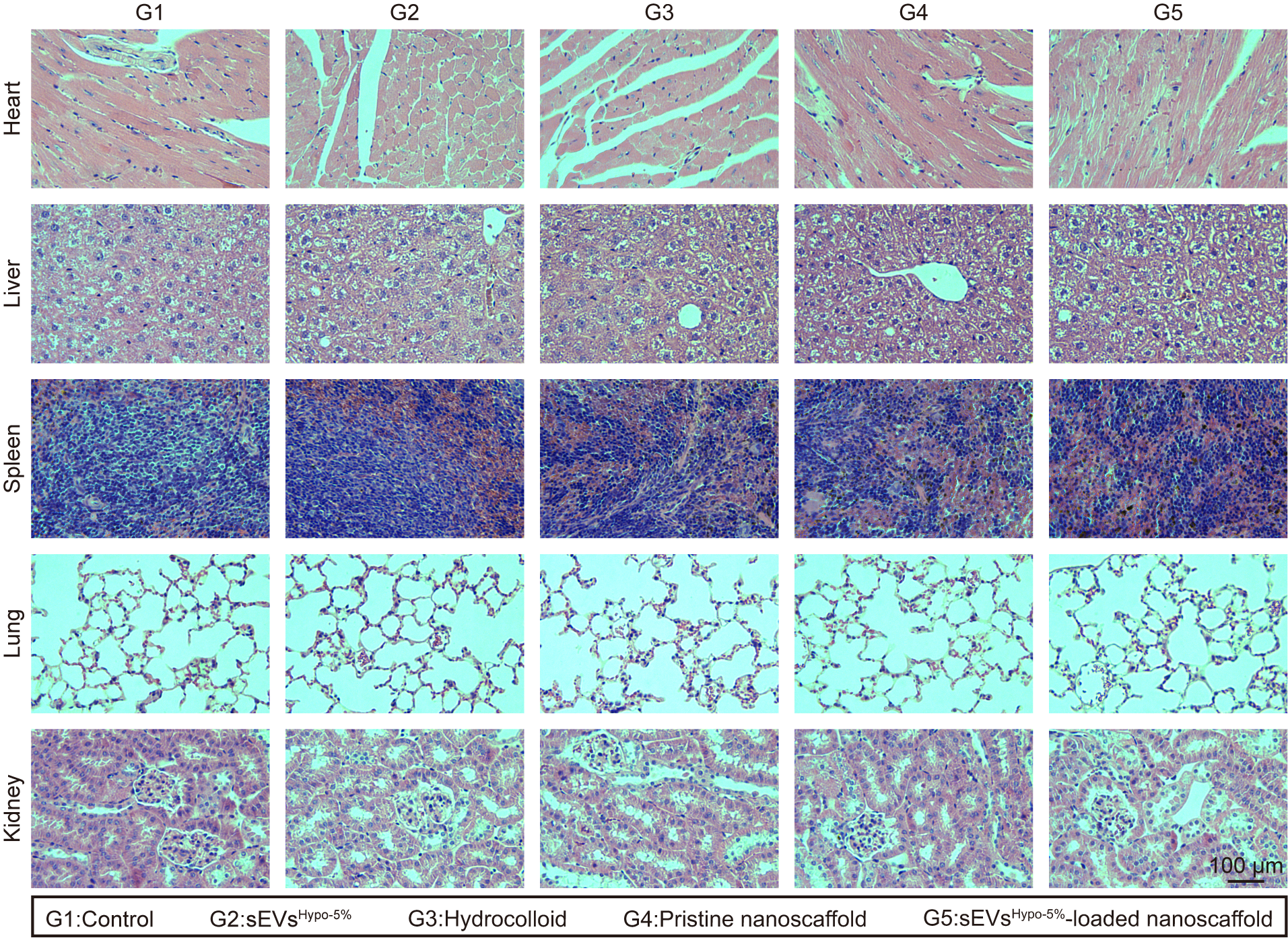

Supplement: Supplementary 1 — Figs. S1 to S15 [file research.1248.f1.zip › S15.tif]

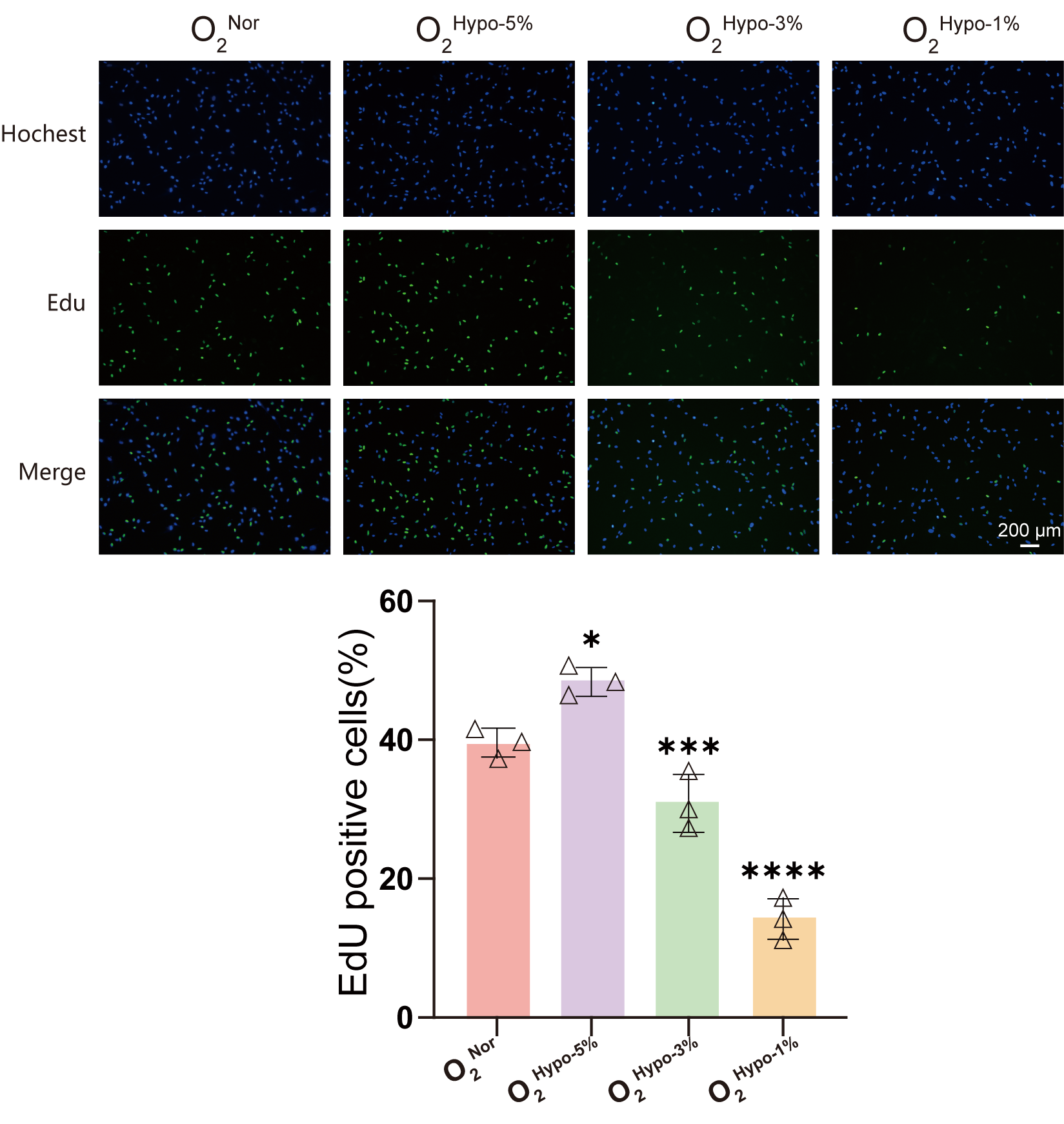

Supplement: Supplementary 1 — Figs. S1 to S15 [file research.1248.f1.zip › S3.tif]

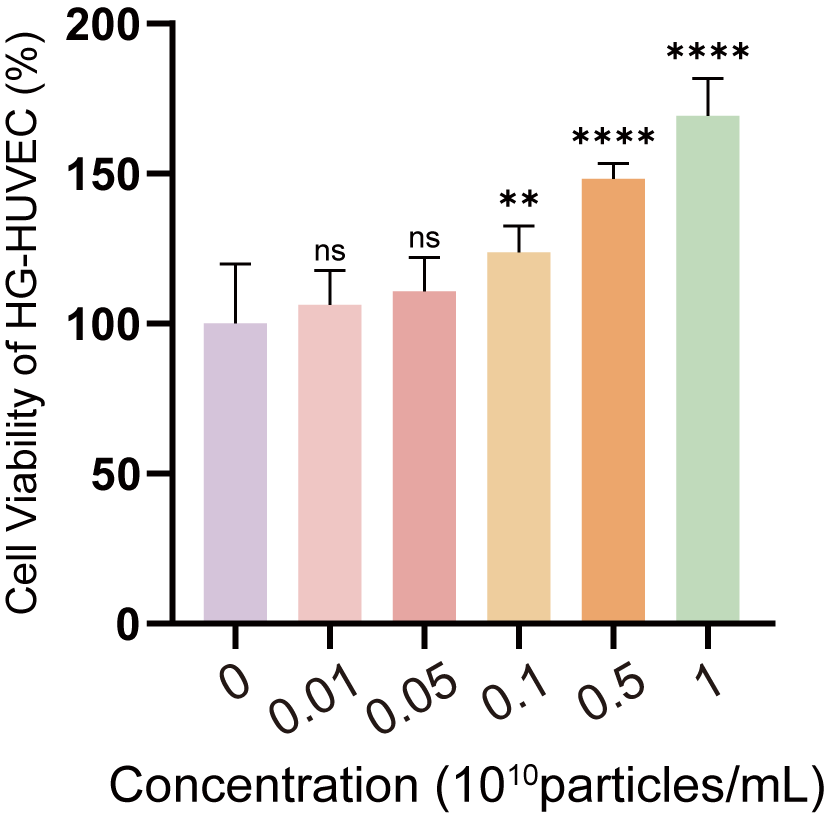

Supplement: Supplementary 1 — Figs. S1 to S15 [file research.1248.f1.zip › S4.tif]

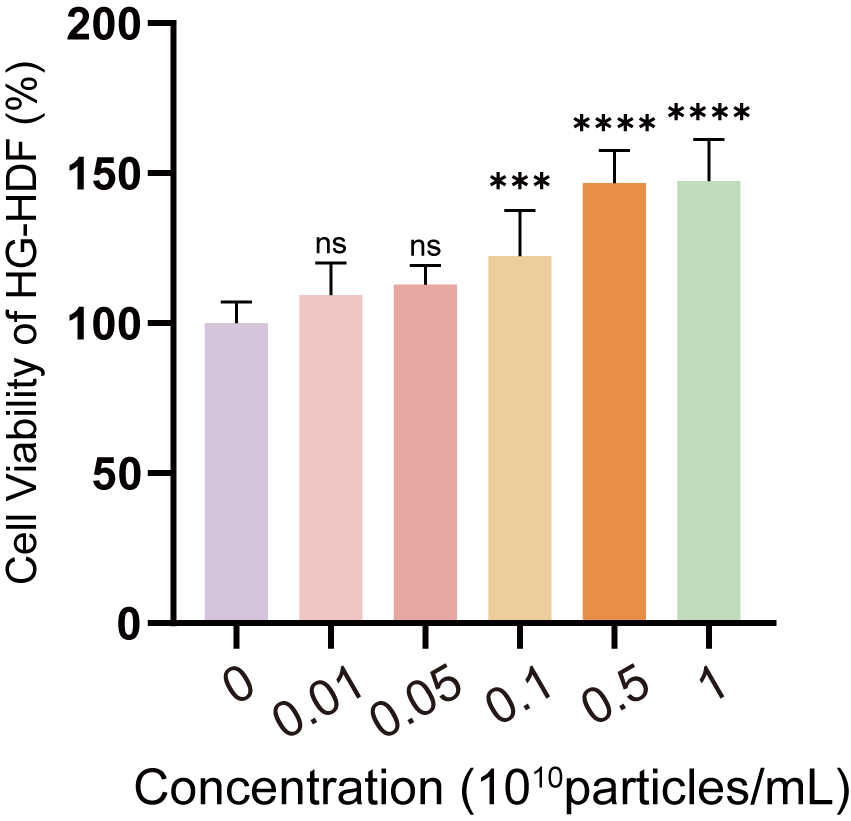

Supplement: Supplementary 1 — Figs. S1 to S15 [file research.1248.f1.zip › S5.tif]

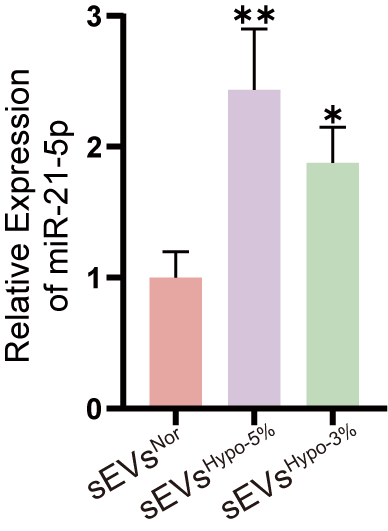

Supplement: Supplementary 1 — Figs. S1 to S15 [file research.1248.f1.zip › S6.tif]

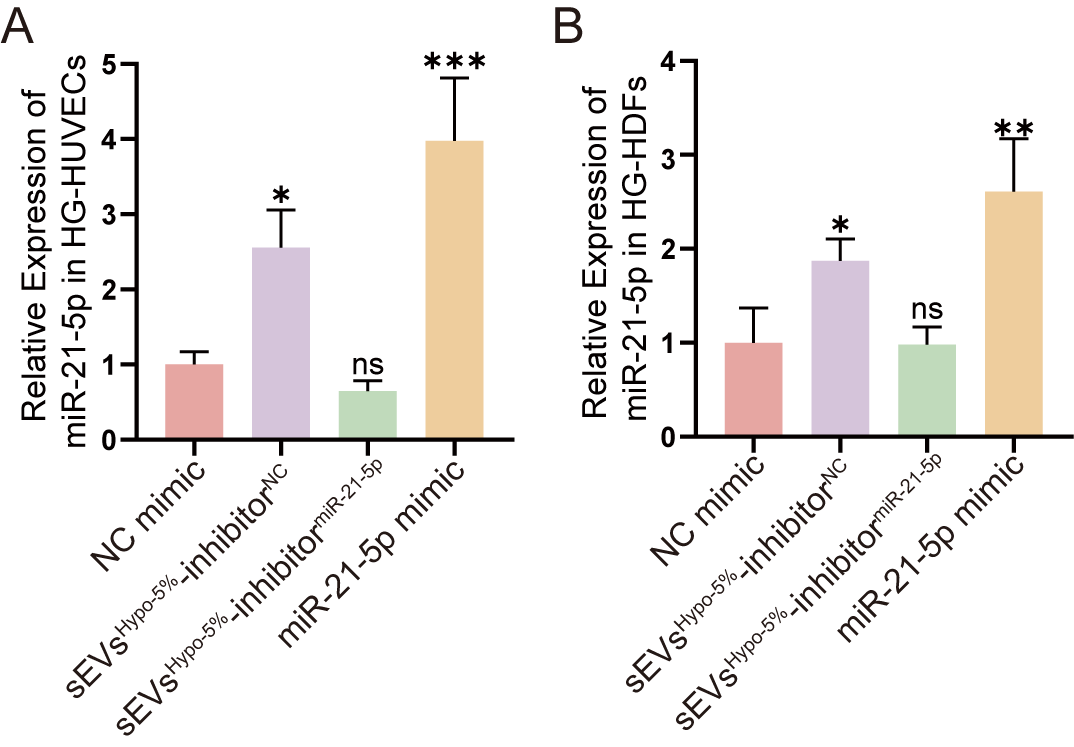

Supplement: Supplementary 1 — Figs. S1 to S15 [file research.1248.f1.zip › S7.tif]

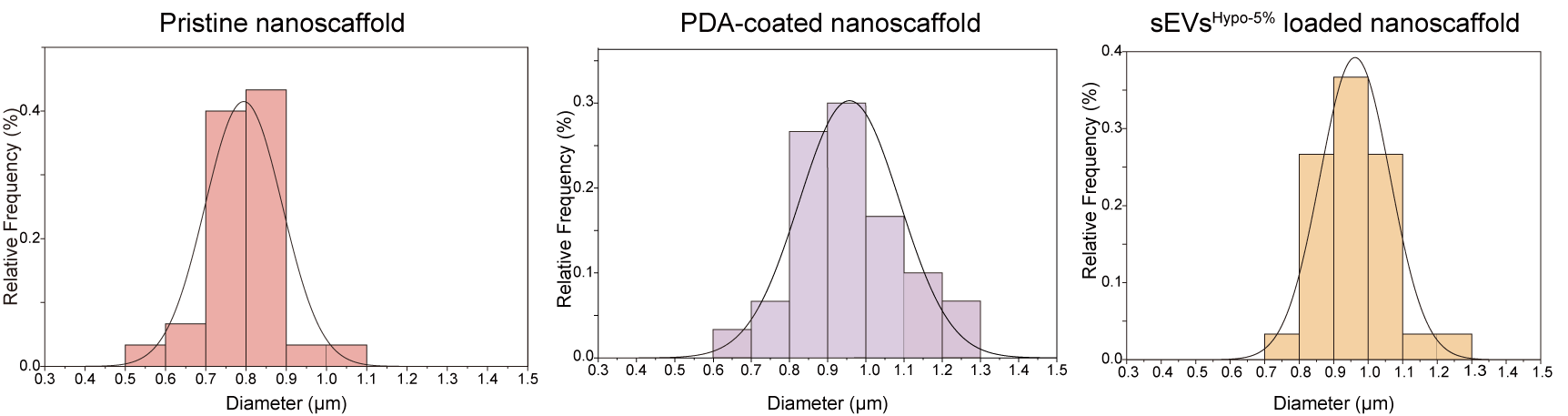

Supplement: Supplementary 1 — Figs. S1 to S15 [file research.1248.f1.zip › S9.tif]
